# Galectin-3: a novel antimicrobial host factor identified in goat nasal mucus

**DOI:** 10.1186/s13567-025-01586-w

**Published:** 2025-07-21

**Authors:** Yichao Ma, Xinming Qin, Jiachen Liu, Shiqi Liu, Ruoyang Lin, Baoyan Meng, Xiaojing Cui, Qian Yang

**Affiliations:** MOE Joint International Research Laboratory of Animal Health and Food Safety, College of Veterinary Medicine, Nanjing Agricultural University, Weigang 1, Nanjing, 210095 Jiangsu China

**Keywords:** Nasal mucus, antimicrobial activity, galectin-3, binding teichoic acids, inhibiting protein synthesis

## Abstract

Respiratory infections caused by pathogenic bacteria pose a rapidly growing public health threat. The nasal mucus layer serves as the first line of defense against pathogen invasion; however, in nasal mucus, the antimicrobial components and their underlying mechanisms remain unclear. Here, we collected nasal mucus from goat nasal mucosal explant models and identified significant antimicrobial activity in the total protein fraction. Subsequent fractionation indicated that proteins < 30 kDa exhibited the most potent bactericidal activity. Nano LC–ESI–MS/MS analysis identified galectin-3 as a key protein with potent activity against Gram-positive bacteria, particularly *Streptococcus suis* (*S. suis*). Galectin-3 targeted teichoic acids on the bacterial surface, disrupting membrane integrity. Additionally, it inhibited the synthesis of three critical bacterial proteins: enoyl-ACP reductase (FabK), carbamate kinase (CK), and small ribosomal subunit protein uS2 (rpsB), thereby destroying bacterial growth and metabolism. In the murine nasal infection model, galectin-3 accelerated the clearance of *S. suis* and alleviated pathological damage caused by the infection. Taken together, our findings provide the first evidence of the direct antimicrobial action of galectin-3 in nasal mucus and elucidate its mechanisms involving bacterial membrane disruption and inhibition of key metabolic proteins. These results highlight galectin-3 as a promising therapeutic target for *S. suis* infections.

**Supplementary Information:**

The online version contains supplementary material available at 10.1186/s13567-025-01586-w.

## Introduction

Respiratory tract bacterial infections represent a significant public health issue worldwide, presenting serious health risks to both humans and animals [1, 2]. The nasal cavity, as the entry point of the respiratory tract, is directly exposed to external pathogens and serves as the primary portal for pathogen invasion into the host [3, 4]. Pathogen invasion of the nasal mucosa can lead to respiratory system lesions and may spread to other organs via the blood or lymph, resulting in localized or systemic infection and damage [5–7]. For instance, the colonization of the nasal mucosa by *Streptococcus pneumoniae*, followed by its dissemination to the ear space, lungs, bloodstream, and meninges, can lead to various diseases, including otitis media, pneumonia, septicemia, and meningitis [8–11]. *Klebsiella pneumoniae*, a primary pathogen associated with community-acquired pneumonia, overcomes the immune defenses of host epithelial cells via its virulence factors, particularly when host immunity is compromised. This bacterium can induce pneumonia that progresses from the upper to the lower respiratory tract and may disseminate through the bloodstream, potentially affecting organs such as the liver, kidneys, heart, and spleen, which can result in sepsis or septic conditions [12, 13]. Consequently, enhancing the nasal mucosal barrier is crucial for defending against infections from respiratory tract pathogens.

The mucus layer in the nasal cavity serves as the initial barrier against respiratory tract pathogens, with various antibacterial proteins significantly contributing to the immune response within nasal mucus [14, 15]. Mucin is among the initial molecules that have been investigated and validated for its antibacterial properties. A dynamic and adjustable polymerization network can be established among its molecules via disulfide bonds, hydrogen bonds, electrostatic interactions, and other mechanisms, serving as a physical barrier [16]. Furthermore, the conventional antibacterial elements found in mucus, such as lysozyme [17], defensin [18], and lactoferrin [19], are crucial in the defense against respiratory infections. Nonetheless, the intricate patterns of respiratory infection and the rising resistance of pathogens are revealing the limitations of the mechanisms of action and defensive efficacy of these conventional immune components [20]. The discovery of novel antibacterial proteins in nasal mucus enhances our understanding of nasal immune defense mechanisms and offers innovative insights for developing effective anti-infection strategies.

Galectin-3, a glycan-binding protein with a conserved carbohydrate recognition domain (CRD) [21], binds β-galactosides on glycoproteins and participates in numerous biological processes [22, 23]. Recent investigations indicate that galectin-3 is significant in host–pathogen interactions. Certain glycoproteins present on the surfaces of bacteria, viruses, fungi, and parasites can bind to galectin-3, modulate the chemotaxis and activation of host immune cells, and contribute to the innate immune response. However, the importance of galectin-3 in nasal mucus and its function in combating bacterial infections remain inadequately explored.

Given the significance of nasal mucus in defense against respiratory pathogens, this study detected galectin-3 in nasal mucus for the first time using Nano LC–ESI–MS/MS technology. The CFU microassay results show that galectin-3 inhibited the growth of Gram-positive bacteria, particularly *Streptococcus suis*. Galectin-3 bound to teichoic acids on the bacterial surface, neutralized the negative charge, increased surface hydrophobicity, and depolarized the membrane potential, with electron microscopy confirming the disruption of bacterial membrane integrity. Furthermore, we investigated the inhibitory effects of galectin-3 on bacterial protein synthesis, elucidating its potential role as a natural antibacterial agent in nasal mucus.

## Materials and methods

### Reagents

His-tag antibody (Proteintech, 1:5000, HRP-66005), galectin-3/LGALS3 antibody (Abclonal, IHC, 1:100; WB, 1:2000; A11198), β-actin antibody (Abways, 1:5000, AB0035), Enhanced ATP Assay Kit (Beyotime, S0027), Bacterial Viability Assay Kit (Beyotime, C2030S), BCA Protein Quantification kit (Vazyme, E112-01), DiOC_2_(3) (Solarbio, 905-96-4), Alkaline phosphatase assay kit (Nanjing Jiancheng,  A059-2-2), recombinant human galectin-3/LGALS3 Protein (ABclonal, RP00057), recombinant Sumo-Flag-His protein (Bioworlde， NBP0540-2), lipoteichoic acid from *Staphylococcus aureus* (MCE，  HY-N9481), and peptidoglycan from *Staphylococcus aureus* (Maclin Reagents, P742432).

### Strains

*Pasteurella multocida* (Serotype D) was prepared and preserved in our laboratory and cultured in tryptone soya broth (TSB). *Streptococcus suis* (ZY05719) was prepared and preserved in our laboratory and cultured in Todd Hewitt Broth (THB). *Staphylococcus aureus* (ATCC25923), *Salmonella typhimurium* (ATCC14028), *and Escherichia coli* (O157:H7) were prepared and preserved in our laboratory and cultured in Luria–Bertani (LB) broth medium.

### Inoculation of pathogenic bacteria in the goat nasal mucosa explant model

This study involved the euthanasia of 6-month-old healthy goats via intravenous administration of pentobarbital sodium at a dosage of 100 mg/kg. The goat's head was severed at the median sagittal plane, and the nasal mucosa was collected along with the turbinate, thereafter immersed in pre-cooled physiological saline containing 5% penicillin–streptomycin solution, 1% amphotericin, and gentamicin. Following the irrigation of the nasal mucosa with antibiotic-infused normal saline three times, the mucosa was delicately stripped from the turbinate using sterile surgical equipment. The isolated nasal mucosa tissue was washed three times with normal saline and then sectioned into uniform 8 mm round tissue blocks using a tissue sampler. The mucosa was positioned flat in a 12-well cell plate, and 300 μL of antibiotic-free complete media was added. Following the overnight culture of the goat explant model, *S. suis* and *Pasteurella multocida* were subjected to centrifugation at 5000 rpm for 10 min, washed twice with PBS, resuspended to 10^6^ CFU/mL, and injected into the goat nasal mucosa explant model using 300 μL in each well. The supernatant of the explant culture model was collected at 3 h, 12 h, and 24 h by colony counts.

### Nasal mucus preparation and total protein extraction

Following a 24 h in vitro culture of the goat nasal explant model, the mucus produced by the model was collected, transferred into a 15 mL centrifuge tube, and subsequently vortexed to ensure thorough mixing. The insoluble portion of the crude mucus was eliminated using centrifugation at 5000 × *g* for 30 min at 4 °C. The total proteins of nasal mucus were obtained using the ammonium sulfate precipitation method, and the protein sample concentration was measured with a BCA Protein Assay Kit.

### The antibacterial activity of nasal mucus protein was detected by CFU microassays

All kinds of pathogenic bacteria cultured in the logarithmic phase were diluted to 10^7^/mL. After 20 μL of diluted bacteria were incubated with 100 μg/mL of nasal mucus protein for 3 h, the co-incubated samples or their dilutions were evenly coated in the corresponding solid culture medium and cultured in a constant temperature incubator at 37 °C for 16 to 18 h to allow visible colonies to grow and count colonies.

### Nano-LC–ESI–MS/MS analysis

Following freeze-drying, nasal mucus protein was redissolved in an aqueous solution containing 2% acetonitrile and 0.5% formic acid. The protein solution underwent reduction with DTT and subsequent alkylation using iodoacetamide. After desalting using a desalting column, the sample underwent digestion with trypsin. The samples were subsequently analyzed using nano-LC-ESI MS/MS. The high-pressure liquid chromatography (HPLC) system (Agilent) is online and connected with a linear ion trap mass spectrometer (LTQ, Thermo). Mobile phase A: 97.5% water, 2% acetonitrile, and 0.5% formic acid. Mobile phase B: 9.5% water, 90% acetonitrile, and 0.5% formic acid. The gradient duration was 60 min, transitioning from 2% Solvent B to 90% Solvent B, in addition to 20 min for sample loading and 20 min for column washing. The mass spectrometer operates in data-dependent mode to obtain MS/MS data through a low-energy collision-induced dissociation (CID) process. The collision energy is 33%, and the charge state is 3. A single full scan with one microscan covering a mass range of 350 amu to 1650 amu was obtained, followed by nine MS/MS scans of the nine most intense ions, each with a full mass range and three microscans. The dynamic exclusion feature was configured with a repeat count of one and an exclusion duration of one minute. The exclusion width measures 4 Da. The mass spectrometric data was utilized to query the UniProt protein database using ProtTech’s ProtQuest software suite.

### Immunohistochemical staining

After dewaxing with xylene, paraffin sections were rehydrated in a graded ethanol series (100%, 95%, 85%, and 75%). The dewaxed and rehydrated sections were incubated in citrate buffer (pH = 6.0) for 30 min for antigen repair. Then, 3% H_2_O_2_ was added to eliminate endogenous peroxidase activity, and the sections were incubated at 37 °C for 1 h. After washing with PBS three times, 5% BSA was applied for 1.5 h to block nonspecific antibody binding at room temperature. Galectin-3 antibody was added and incubated overnight at 4 °C. After washing with PBS three times, the corresponding biotinylated secondary antibody was incubated for 1.5 h. Following three PBS washes, SABC was added, and incubation continued at 37 °C for 1 h. After another three PBS washes, DAB color-developing solution was applied, and the color development time was controlled under a microscope. Finally, hematoxylin was used for counterstaining the nuclei, and the slides were mounted.

### Expression and purification of galectin-3

The galectin-3 gene was amplified using cDNA derived from goat nasal mucosal tissue and cloned into the pET-22b plasmid. The recombinant plasmid was transformed into *E. coli* competent BL21 cells, and expression was induced with 0.2 mM isopropyl-β-D-thiogalactopyranoside (IPTG) at OD600 = 0.5. The cultures were then grown overnight at 25 °C. The bacterial cell pellets were harvested by centrifugation and resuspended in lysis buffer (50 mM Tris–HCl pH 7.5, 150 mM NaCl, 10 mM imidazole). Cell disruption was performed under cold conditions using high-pressure homogenization, and the mixture was centrifuged at 12 000 rpm for 20 min at 4 °C to collect the supernatant. Galectin-3 was purified using an Ni–NTA column on an ÄKTA system.

### Determination of minimum inhibitory concentration (MIC)

The MIC was determined using the microdilution method. Galectin-3, with an initial concentration of 800 μg/mL, was serially diluted and added to a 96-well plate, with 100 μL per well. Bacteria in the logarithmic phase of growth were diluted to 1 × 10^5^ CFU/mL, and 100 μL of the diluted bacterial suspension was added to each well of the 96-well plate. The plate was incubated at 37 °C for 12 h in a constant-temperature incubator, after which resazurin indicator was added and the incubation continued for an additional 2 h. The MIC was defined as the lowest drug concentration that prevented a blue to pink color change.

### Time-kill kinetics assay

*S. suis* cells cultured to the logarithmic phase were diluted to 10^6^/mL in THB medium and treated with galectin-3 at concentrations of 0.25 × MIC, 0.5 × MIC, and 1 × MIC. The control group was treated with an equal volume of PBS. The cultures were incubated at 37 °C in a shaking incubator at 180 rpm. At time points 0 h, 2 h, 4 h, 6 h, 8 h, 10 h, and 12 h, aliquots were collected from the corresponding tubes and plated for colony counting. The inhibition curve was constructed by plotting the logarithmic value of viable bacterial count against time.

### Bacterial binding assay

In the enzyme-linked immunosorbent assay (ELISA) analysis, *S. suis* was diluted with coating buffer (15 mM Na_2_CO_3_ and 35 mM NaHCO_3_ [pH = 9.6]) and coated onto a 96-well plate with a volume of 100 μL per well (1 × 10^7^ CFU/mL), incubated overnight at 4 °C. The plate was blocked with 5% BSA for 1 h. After each step, unless otherwise stated, the plate was washed three times with TBST. All ELISA steps were performed at 37 °C. Galectin-3 at a concentration of 100 μg/mL was added, with BSA as the control. The plate was incubated at 37 °C for 2 h, washed three times with TBST to remove unbound galectin-3, and then incubated with HRP-conjugated mouse anti-His monoclonal antibody for 1 h. After three washes with TBST, TMB substrate solution was added for color development, incubating for 15 min. The reaction was terminated by adding 2 mol/L H_2_SO_4_, and the absorbance was measured at 450 nm.

In the sugar-binding analysis, peptidoglycan (PGN), lipoteichoic acid (LTA), and wall teichoic acid (WTA) were coated onto a 96-well plate at 4 °C overnight (100 μL per well, 100 μg/mL). The remaining steps were the same as those in the bacterial binding assay described above.

For western blot analysis, *S. suis* in the logarithmic growth phase was collected by centrifuging at 5000 rpm for 10 min, washed three times with PBS, and resuspended in PBS at a concentration of 2.0 × 10^7^ CFU/mL. A 500 μL aliquot of bacteria was incubated with 500 μL of purified galectin-3 (25 μg/mL) at room temperature for 1 h with rotation. Bacteria treated with Sumo-Flag-His protein served as the control. After incubation, the bacteria were washed three times with PBS and finally eluted with a 5% SDS solution. The eluted solution was analyzed by western blot to assess the binding effect.

For the bacterial agglutination test, *S. suis* was cultured to the logarithmic phase, centrifuged at 5000 rpm for 10 min, and washed three times with PBS. The bacteria were labeled with CFSE and then incubated with galectin-3 at 37 °C for 2 h. Aggregation of bacteria was observed under a fluorescence microscope.

### Effect of galectin-3 on *S. suis* protein synthesis

*S. suis* cultured to the logarithmic phase was diluted to 10^7^/mL in THB medium and treated with 25 μg/mL galectin-3, while the control group received an equal volume of PBS. The cultures were incubated at 37 °C in a thermostatic shaker at 180 rpm for 2 h. After centrifugation, the concentrations of the samples were measured using a turbidity meter, and the bacteria were diluted with PBS to achieve the same bacterial concentration. The samples were then centrifuged again to collect the bacterial pellets. The pellets were resuspended in 75 μL of PBS, and 4 × SDS-PAGE loading buffer was added and mixed. The samples were boiled for 10 min and analyzed by 10% SDS–polyacrylamide gel electrophoresis (SDS–PAGE). The gel underwent staining with Coomassie Brilliant Blue. Protein bands exhibiting differential expression were excised for analysis using nano LC–ESI–MS/MS.

### Animal experiments

Six-week-old female BALB/c mice were purchased from the Comparative Medicine Center of Yangzhou University. The mice were kept in the Laboratory Animal Center of Nanjing Agricultural University, maintained under specific-pathogen-free conditions. The ambient temperature was controlled at 22 ± 1 °C, and humidity ranged from 30 to 70%, with a light cycle of 12 h light/12 h dark cycle. Additionally, the facility ensured adequate ventilation and allowed unrestricted access to food and water. Subsequent tests may be conducted after a 3-day adaptation period. To assess the efficacy of galectin-3 in clearing *S. suis* in vivo, 84 mice were randomly divided into seven groups: a blank control group, a galectin-3 control group, an *S. suis* infection group, a high-dose galectin-3 protection group (4 mg/kg), a low-dose galectin-3 protection group (2 mg/kg), a lactose and galectin-3 co-incubation group, and an *S. suis* pre-incubated with galectin-3 group. Mice were intranasally inoculated with 70 μL PBS or 2 × 10^9^ CFU of *S. suis*. Mice in the galectin-3 protection group, lactose inhibition group, and galectin-3 pre-treatment group received intranasal administration of galectin-3 2 h prior to and 2 h following *S. suis* infection. In the lactose inhibition group, galectin-3 was incubated with 25 mM lactose for 30 min before administration. In the galectin-3 pre-treatment group, *S. suis* was pre-treated with galectin-3 for 2 h before use. Blood and homogenates of brain and lungs were collected from the mice on days 1 and 3 post-infection for colony counting. Frozen tissue samples from the nose, lungs, and brain were also collected for subsequent analysis, and tissues were fixed in paraformaldehyde and processed into paraffin sections for histopathological analysis and *S. suis* bacterial load detection.

### Statistical analysis

The analysis of data was conducted utilizing GraphPad Prism version 9.4.1, with results expressed as means ± standard deviation (SD). One-way ANOVA was utilized to assess significant differences across multiple groups, while t-tests were applied to evaluate differences between pairs of groups. The thresholds for significance were established as follows: NS indicates no significance, *P < 0.05, **P < 0.01, and ***P < 0.001.

## Results

### Nasal mucus proteins possess antimicrobial activities

**Figure 1:**
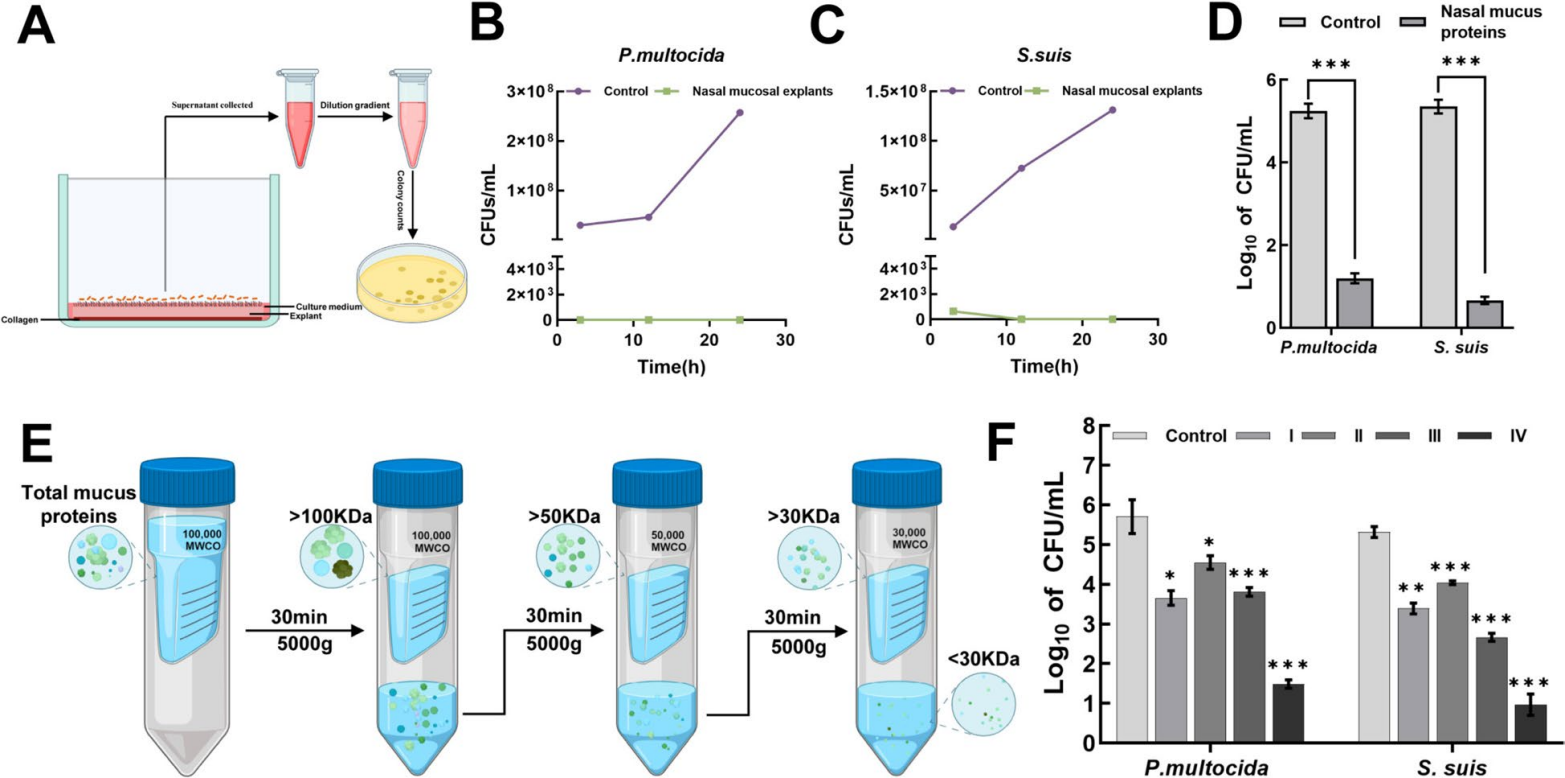
**Antibacterial Activity of Nasal Mucus Proteins. A** Schematic diagram of pathogen inoculation in the goat nasal mucosa explant model (Created in BioRender). The goat nasal mucosa explant model was cultured overnight and subsequently inoculated with *Pasteurella multocida* **(B)** and *S. suis* **(C)** at a concentration of 10⁶ CFU/mL. Supernatants were collected at 3 h, 12 h, and 24 h post-inoculation for bacterial quantification via colony counting. **D** The antibacterial activity of total nasal mucus proteins was evaluated through CFU microassays. Nasal mucus proteins were isolated using ammonium sulfate precipitation. Freshly cultured *Pasteurella multocida* and *S. suis* in the logarithmic phase were diluted to 10⁷ CFU/mL. A 20 μL bacterial suspension was incubated with 50 μL of total nasal mucus protein (100 μg/mL) or PBS in 1.5 mL microcentrifuge tubes at 37 °C for 3 h. The complete sample or its dilutions were uniformly plated on TSA or THA agar plates and incubated at 37 °C for 16–18 h to facilitate visible colony formation for counting. **E** The schematic diagram illustrates the separation of nasal mucus proteins into four molecular weight fractions: > 100 kDa, 100–50 kDa, 50–30 kDa, and < 30 kDa, utilizing ultrafiltration centrifugation (Created in BioRender). **F** The antibacterial activity of nasal mucus proteins (I: > 100 kDa; II: 100–50 kDa; III: 50–30 kDa; and IV: < 30 kDa) across various molecular weight fractions was assessed through CFU microassays. All data presented as mean ± SD from three independent experiments. Statistical significance was performed via one-way ANOVA. NS, not significant; *P < 0.05; **P < 0.01; ***P < 0.001.

*Pasteurella multocida* and *S. suis* were introduced into the in vitro long-term culture model of goat nasal mucosa explants, respectively. The prolongation of the inoculation period resulted in a substantial reduction in the survivability of the two pathogenic bacteria, with no live bacteria detectable up to 24 h (Figures 1B and 1C). Because the goat nasal explant model secreted a lot of mucus, the ammonium sulfate precipitation method was employed to extract the total proteins of nasal mucus, which was then utilized to measure the antibacterial activity of the mucus using the colony counting method. Figure 1D illustrates that total nasal mucus protein exhibited resistance to *Pasteurella multocida* and *S. suis*. Specifically, a concentration of 100 μg/mL of total nasal mucus proteins resulted in reductions of 4.05 and 4.69 log for *Pasteurella multocida* and *S. suis*, respectively, when compared to the blank control. Additionally, the total nasal mucus proteins were divided into four fractions: > 100 KDa, 100–50 KDa, 50–30 KDa, and < 30 KDa. Figure 1F illustrates that the mucus proteins of various fractions exhibited specific antibacterial effects, with the fraction of < 30 kDa exhibiting the most significant effect. The results indicate that nasal mucus proteins exhibit a significant bactericidal effect.

### Screening and identification of antimicrobial active compounds in nasal mucus

**Figure 2:**
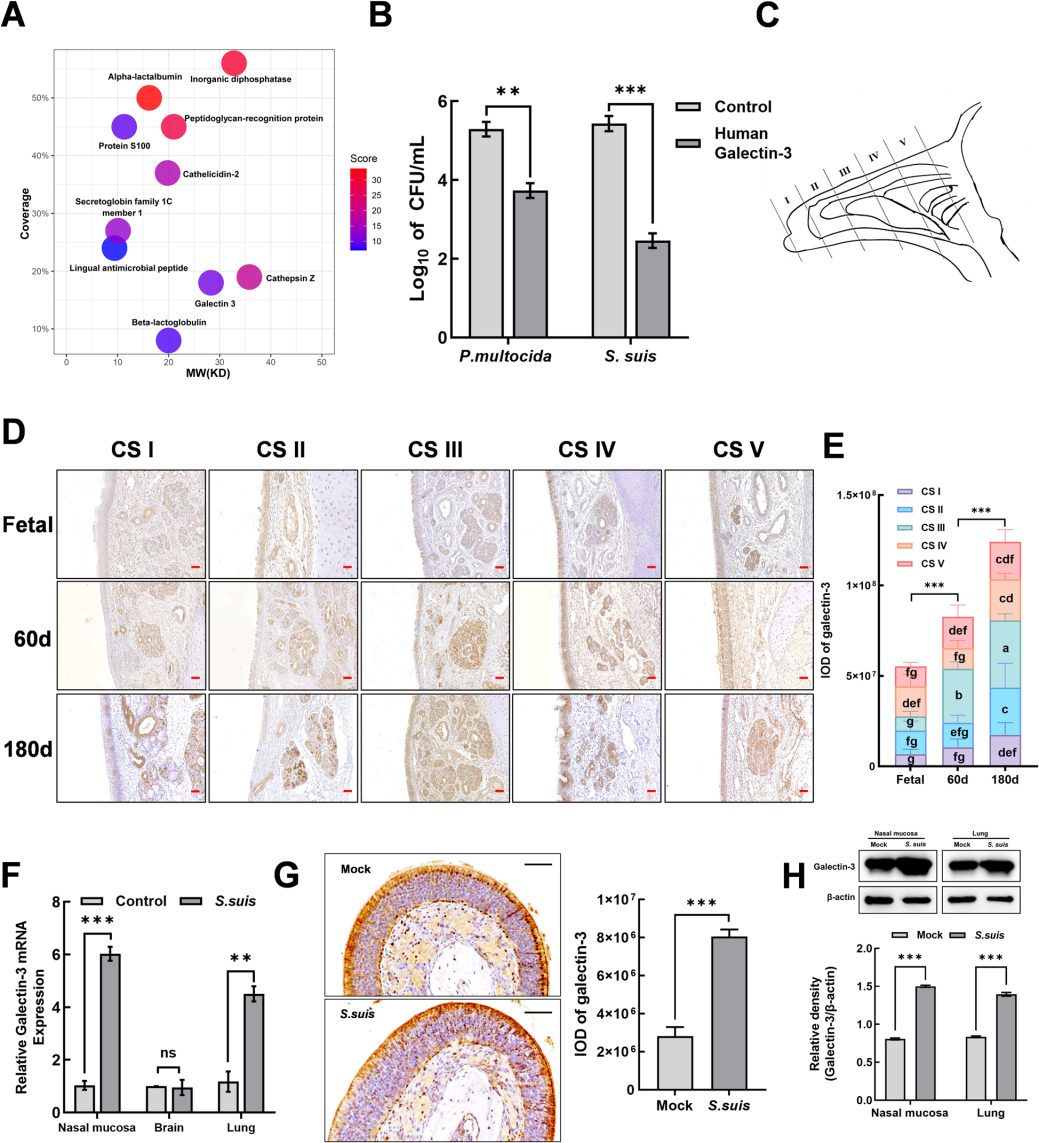
**Identification of antibacterial components in nasal mucus. A** Protein components in the < 30 kDa fraction were identified through nano LC–ESI–MS/MS analysis. **B** The antibacterial effects of commercial recombinant human galectin-3 against *Pasteurella multocida* and *S. suis* were evaluated using CFU microassays. **C** Schematic diagram of the nasal cavity regions in goats. **D** Immunohistochemical staining was used to detect the distribution of galectin-3 in the vestibular, respiratory, and olfactory areas of the nasal cavity in fetal, 60-day-old, and 180-day-old goats. **E** During the statistical analysis of the immunohistochemical staining results for galectin-3, differences are indicated by different letters. Letters above the graphs denote statistical significance, with treatments sharing a common letter not showing significant differences from each other. **F–H** Investigation of galectin-3 expression in mice following nasal infection with *S. suis*. Six-week-old mice were intranasally infected with *S. suis* at a concentration of 2 × 10⁹ CFU/mL. Nasal tissues were collected 24 h post-infection, embedded in paraffin, and sectioned for immunohistochemical analysis of galectin-3 distribution. **(G)**. Additionally, frozen samples from the nasal cavity, brain and lungs were collected for RT-qPCR **(F)** and western blot **(H)** analysis of galectin-3 expression levels. Data are expressed as mean ± SD from three independent experiments. One-way ANOVA was utilized to assess statistical significance. NS, not significant; *P < 0.05; **P < 0.01; ***P < 0.001.

In order to further screen and identify novel antibacterial active proteins in nasal mucus, we further detected the components of < 30 kDa using nano-LC–ESI–MS/MS. Galectin-3 was chosen as a candidate protein based on its size, peptide coverage, secretability, and subcellular localization (Figure 2A). The raw data from the nano-LC–ESI–MS/MS analysis shown in Figure 2A are available in Additional file 1. Galectin-3 was selected for further investigation among the candidate proteins, based on molecular size, peptide coverage, secretion potential, and the current state of research. In antimicrobial experiments, 100 μg/mL of recombinant human galectin-3 protein diminished *Pasteurella multocida* and *S. suis* by 1.56 and 2.97 log, respectively, in comparison to the control (Figure 2B). Furthermore, the distribution of galectin-3 in the nasal mucosa of goats was observed by immunohistochemical staining. The nasal cavity of the goat is segmented into vestibular (CSI and CSII), respiratory (CSIII and CSIV), and olfactory (CSV) regions (Figure 2C). Galectin-3 was localized in the nasal epithelium and submucosal gland, with the highest secretion observed in the respiratory region (Figure 2D). Moreover, intensity plot analysis of immunohistochemistry staining results revealed the age-dependent increase of galectin-3 in the nasal mucosa of goats (Figure 2E). In addition, galectin-3 expression was elevated as demonstrated by RT-qPCR (Figure 2F), immunohistochemistry (Figure 2G), and western blot (Figure 2H) in mice following nasal administration of *S. suis*, suggesting that galectin-3 may be significant in the antibacterial immunity.

### Purification and antibacterial activity detection of recombinant goat galectin-3

**Figure 3:**
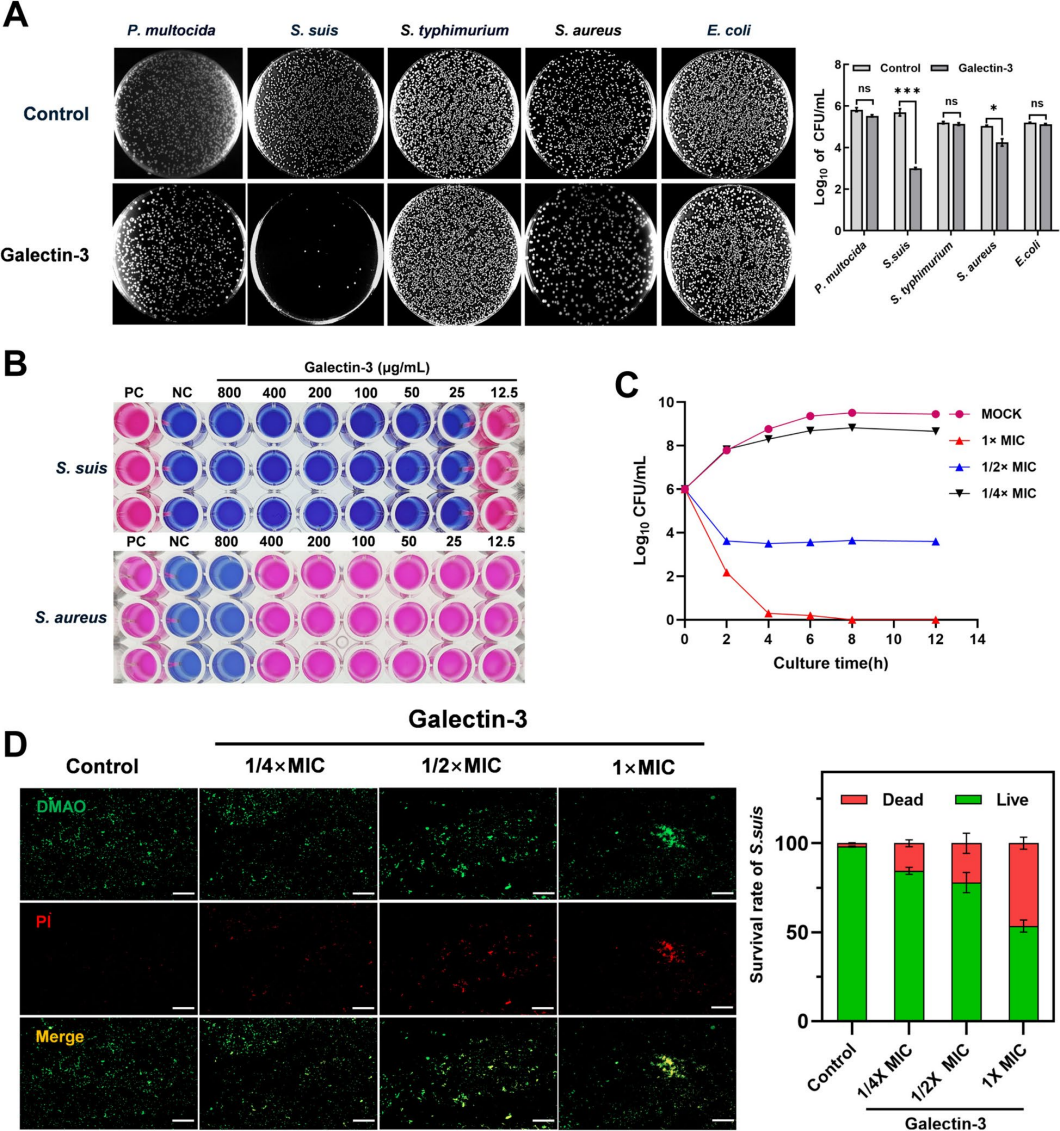
**Antibacterial activity of recombinant goat galectin-3. A** The antibacterial efficacy of recombinant goat galectin-3 against *Pasteurella multocida*, *S. suis*, *Salmonella typhimurium*, *Staphylococcus aureus*, and *Escherichia coli* was detected by CFU microassay. **B** The minimum inhibitory concentration (MIC) of galectin-3 against *S. suis* and *Staphylococcus aureus* was determined using the microdilution method. Galectin-3 (initial concentration = 800 μg/mL) was serially diluted and added to a 96-well plate, followed by the addition of 100 μL of 10^5^ CFU/mL bacteria. After 6 h of incubation, a color change was observed upon the addition of the indicator dye, resazurin. **C** Time-kill kinetics of galectin-3 against *S. suis* in the logarithmic phase was diluted to 10⁶ CFU/mL and treated with 0.25 × , 0.5 × , and 1 × MIC of galectin-3. Colony counts were performed at 0 h, 2 h, 4 h, 6 h, 8 h, 10 h, and 12 h. **D** Effect of galectin-3 on *S. suis* viability using a bacterial viability assay kit. Data are presented as mean ± SD derived from three independent experiments. One-way ANOVA was employed to evaluate statistical significance. NS, not significant; *P < 0.05; **P < 0.01; ***P < 0.001.

To investigate the antibacterial activity of galectin-3, we cloned and constructed prokaryotic expression vectors (Additional files 2A and 2B) for galectin-3, followed by the expression and purification of goat galectin-3. Coomassie blue staining (Additional file 2C) and western blot (Additional file 2D) demonstrate a single positive band approximately 28 kDa in size. Subsequently, we evaluated the inhibitory effect of galectin-3 on several pathogenic bacteria, including *Pasteurella multocida*, *S. suis*, *Staphylococcus aureus*, *Salmonella typhimurium*, and *Escherichia coli.* Figure 3A indicates that galectin-3 exhibited the most significant inhibitory effect on Gram-positive bacteria, particularly *S. suis*, in comparison to Gram-negative bacteria. In the minimum inhibitory concentration (MIC) assay (Figure 3B) utilizing resazurin as the indicator, at concentrations of 25 μg/mL for *S. suis* and 800 μg/mL for *Staphylococcus aureus*, the proliferation of both pathogenic bacteria was entirely suppressed, resulting in a dark blue culture medium. To further assess the bactericidal activity of galectin-3 against *S. suis*, time kill curves were established. Figure 3C illustrates that *S. suis* was effectively eliminated in a dose-dependent manner, with a reduction to undetectable levels after 6 h of incubation when exposed to 1 × MIC galectin-3. In addition, we used the Bacterial Viability Assay Kit to stain *S. suis* treated with galectin-3. Figure 3D illustrates that as the concentration of galectin-3 increased, the proportion of PI fluorescence intensity correspondingly rose, achieving 50% at a concentration of 25 μg/mL.

### Galectin-3 binds and disrupts the integrity of *S. suis* cell membrane

**Figure 4:**
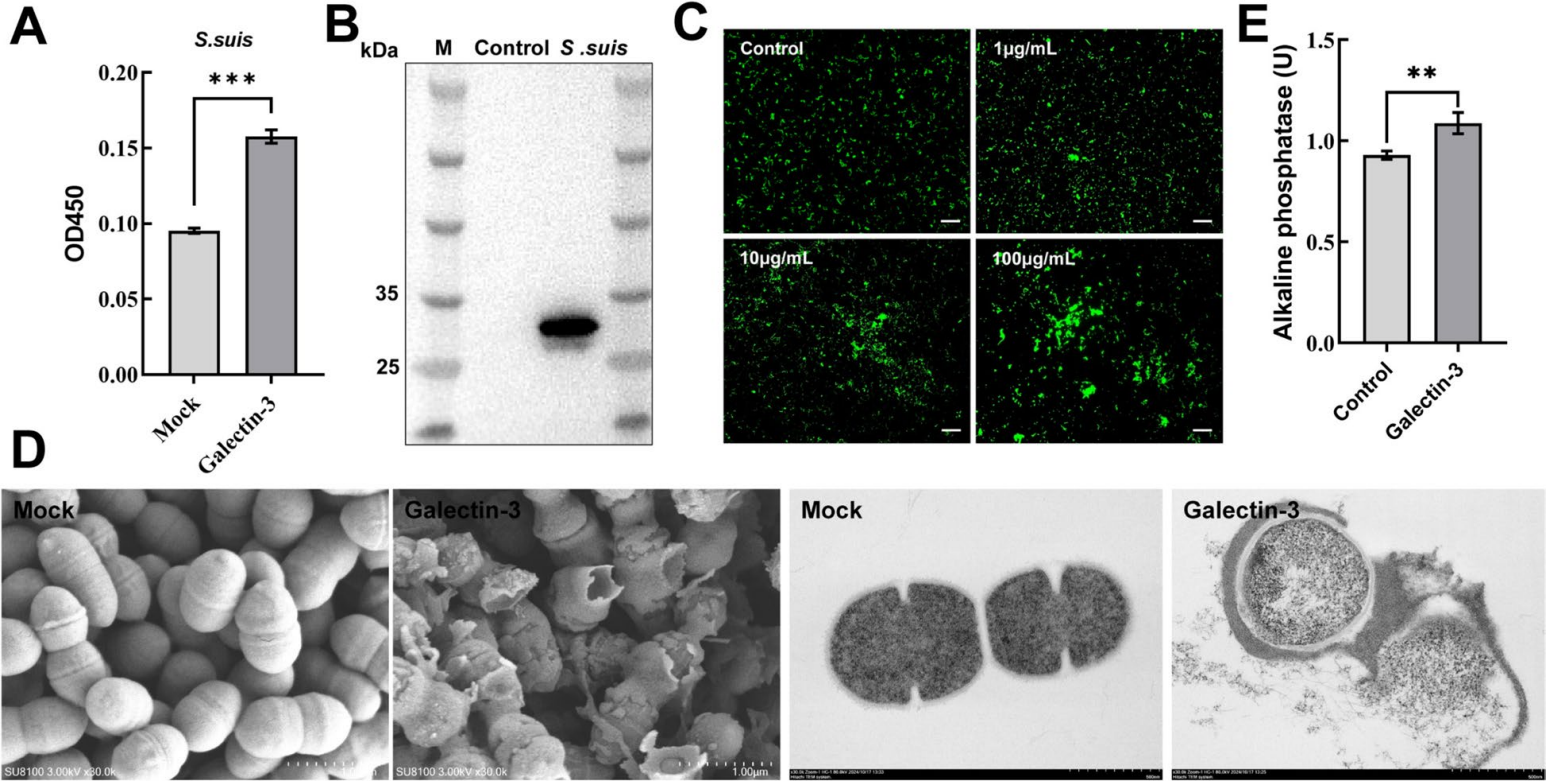
**Galectin-3 binds to the bacterial membrane and disrupts the integrity of *S. suis.*** **A** Quantitative assessment of galectin-3 binding to *S. suis* by ELISA. *S. suis* (100 μL, 10⁷ CFU/mL) was coated onto a 96-well plate and incubated with 100 μg/mL galectin-3 for 2 h. The binding of galectin-3 to *S. suis* was detected using His-tag antibodies. **B** Confirmation of galectin-3 association with *S. suis* via western blot analysis. *S. suis* cultured to the logarithmic phase was incubated with 25 μg/mL galectin-3 at room temperature with a rotation for 1 h. After washing, the bacterial pellet was analyzed by western blot to detect the presence of galectin-3 on the bacterial surface. **C** Visualization of galectin-3-induced bacterial aggregation. *S. suis* labeled with CFSE was incubated with galectin-3 (1, 10, and 100 μg/mL) at 37 °C for 2 h. Bacterial aggregation was observed under a fluorescence microscope. **D** Scanning electron microscopy (SEM) and transmission electron microscopy (TEM) were used to examine the ultrastructural effects of galectin-3 on *S. suis*. **E** Quantitative analysis of alkaline phosphatase (ALP) activity in the supernatant of *S. suis* treated with galectin-3. The data are presented as mean ± SD derived from three independent experiments. One-way ANOVA was employed to evaluate statistical significance. NS indicates no significance; *P < 0.05; **P < 0.01; ***P < 0.001.

Given that galectin-3 possesses a carbohydrate-binding site, we investigated its potential to bind sugar molecules present on the bacterial surface. ELISA (Figure 4A) and western blot (Figure 4B) analyses demonstrate that galectin-3 is capable of binding to *S. suis*. Galectin-3 demonstrates the ability to agglutinate *S. suis*, with a noticeable increase in agglutination correlating with higher concentrations of galectin-3 (Figure 4C). Then, we further evaluated the influence of galectin-3 on the ultrastructure of *S. suis* by scanning electron microscopy (SEM) and transmission electron microscopy (TEM) (Figure 4D). The SEM results indicate that the surface of *S. suis* in the blank control group was smooth, round or ovoid, and arranged in a chain-like morphology. In the galectin-3 treated group, the surface of *S. suis* was rough and unevenly wrinkled, and the cell membrane was severely deformed and ruptured. The TEM data indicate that *S. suis* in the blank control group had complete and smooth-shaped cells with clear membranes and homogeneity of cell matrix. In contrast, the *S. suis* treated with galectin-3 exhibited significant deformation, with a blurred membrane boundary and observable leakage of cytoplasmic contents around the cells. Alkaline phosphatase (ALP) is a significant enzyme situated between the cell wall and the cell membrane. Upon damage to the cell wall, ALP is released. Consequently, ALP serves as a significant indicator for assessing cell wall integrity. Figure 4E illustrates that, relative to the blank control group, the release of ALP increased by 1.18 times following 2 h of galectin-3 treatment. These data indicate that galectin-3 binds to and disrupts the integrity of the *S. suis* cell membrane, thereby exhibiting an antibacterial function.

### Lactose could competitively inhibit the killing effect of galectin-3 on *S. suis*

**Figure 5:**
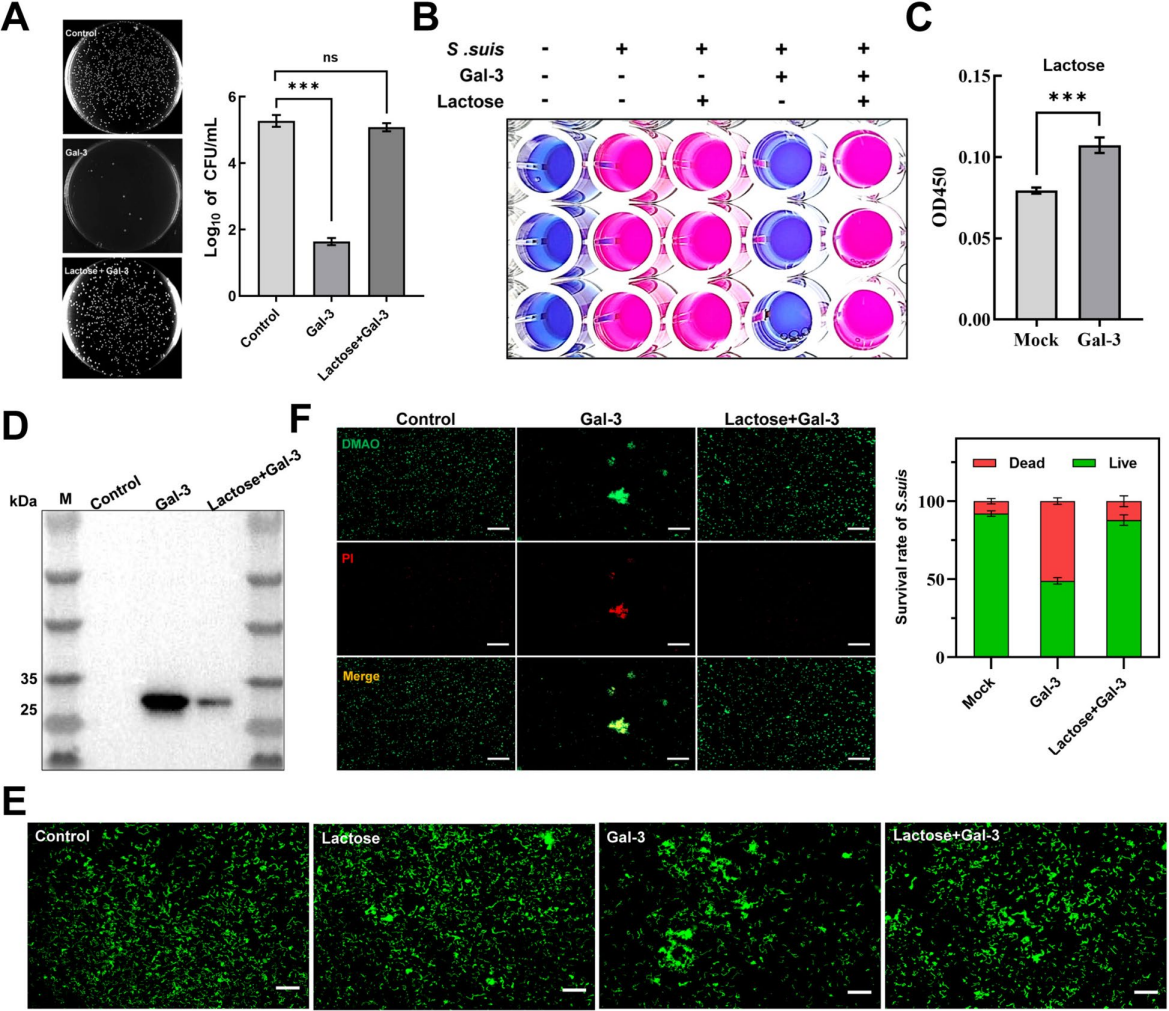
**Galectin-3 demonstrates aggregation and bactericidal properties toward *S. suis* in a manner dependent on β-galactosides. A** The impact of lactose on the antibacterial properties of galectin-3 was assessed through the CFU microassay. *S. suis* in the logarithmic phase (20 μL, 10⁷/mL) were incubated with 50 μL of galectin-3, galectin-3 combined with lactose, or PBS for 3 h and then quantified by counting colony number. **B** The influence of lactose on the anti-*S. suis* efficacy of galectin-3 was evaluated by the microdilution method. **C and D** The influence of lactose on the interaction of galectin-3 with *S. suis* was assessed by ELISA and western blot, respectively. **E** *S. suis* in the logarithmic phase, labeled with CFSE, were incubated with PBS, lactose, galectin-3, or a combination of galectin-3 and lactose for 2 h. Fluorescence microscopy was used to observe bacterial aggregation subsequent to the blockade of the carbohydrate recognition domain of galectin-3 by lactose. **F** A bacterial viability assay kit was employed to evaluate the impact of lactose on the antibacterial efficacy of galectin-3. Statistical significance was performed via one-way ANOVA. NS, not significant; *P < 0.05; **P < 0.01; ***P < 0.001.

To further investigate whether galectin-3 exerts its antibacterial effect by binding to the Galβ (1–4) GlcNAc binding site on bacterial surface sugars, we added lactose, which has a similar binding site (Galβ (1–4) Glc), to competitively bind galectin-3. The CFU microassays (Figure 5A) and MIC test (Figure 5B) findings indicate that lactose decreased the bactericidal effect of galectin-3 on *S. suis*, with 25 mM lactose entirely abolishing the antibacterial activity of galectin-3 against *S. suis*. Moreover, ELISA (Figure 5C) and western blot analysis (Figure 5D) indicate that lactose inhibited the binding of galectin-3 to *S. suis*. As shown in Figure 5E, lactose inhibited the agglutination of galectin-3 to *S. suis*. The results of bacterial viability staining (Figure 5F) indicate that the addition of 25 mM lactose restored the proportion of PI-positive bacteria to levels comparable to the blank control. In addition, the addition of lactose blocked the ultrastructural alterations of *S. suis* caused by galectin-3 (Additional file 3). The results described above show that galectin-3 must bind to the sugars on the bacterial surface to exert its bactericidal effect. Given the high sequence and structural similarity of the CRD of galectin-3 among different species (Additional files 4A and 4B), we assessed the bactericidal activity of porcine and human galectin-3 against *S. suis*. The results demonstrate that both species displayed antimicrobial properties, with MIC values of 25 and 50 μg/mL, respectively (Additional files 4F and 4G). Additionally, considering that pigs are natural hosts of *S. suis*, we compared the expression levels of galectin-3 in the nasal cavities of both pigs and goats. Immunohistochemistry (Additional files 4C and 4D) and ELISA (Additional file 4E) analyses indicate no significant difference in galectin-3 expression between 60-day-old pigs and goats.

### Galectin-3 exerts bactericidal effects by binding to the teichoic acids on the surface of *S. suis*

**Figure 6:**
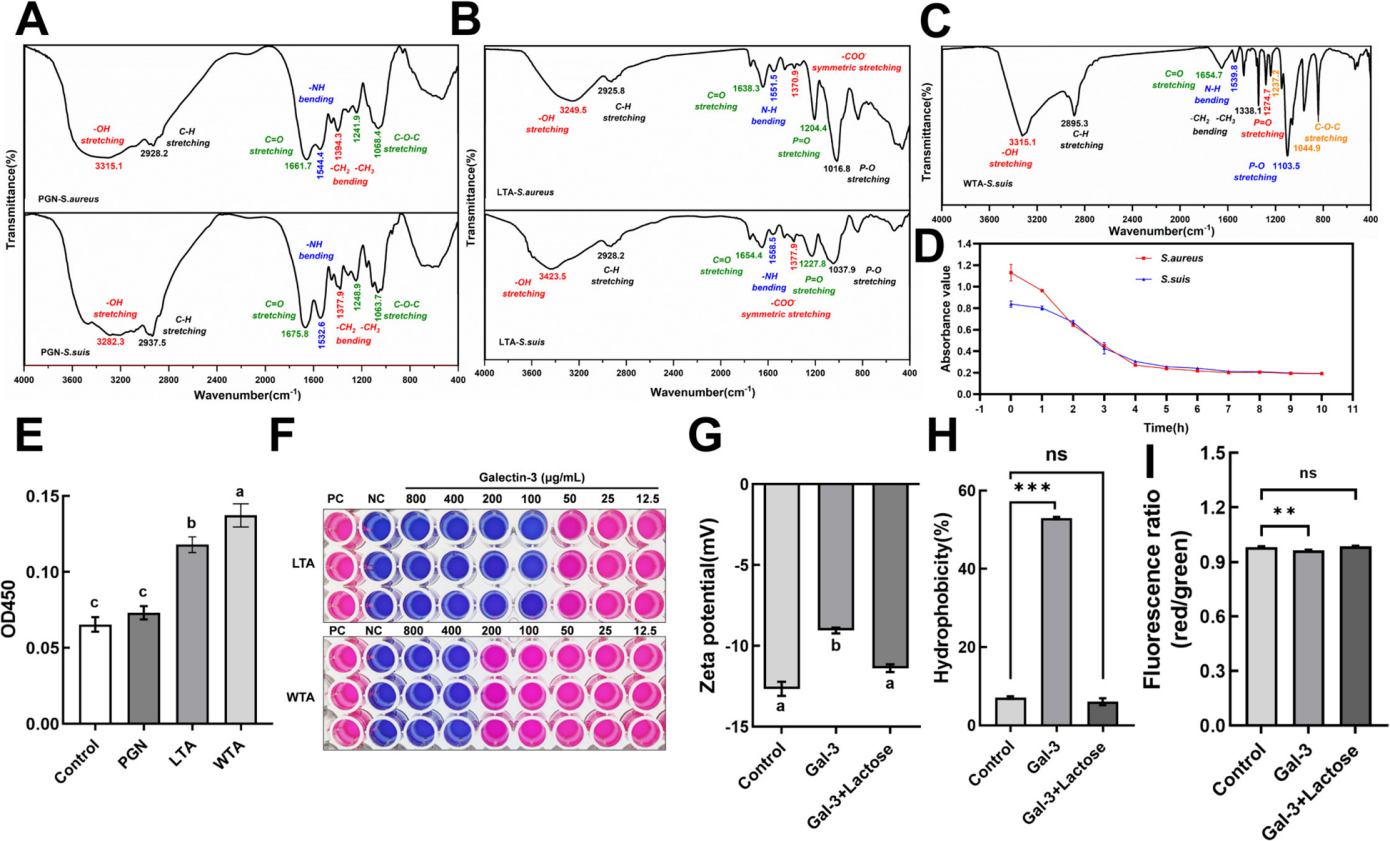
**Galectin-3 exerts antibacterial activity by binding to the teichoic acids. A-C** Fourier transform infrared (FTIR) spectrum analysis of peptidoglycan (PGN), lipoteichoic acid (LTA), and wall teichoic acid (WTA). **D** Lysozyme digestion test of PGN. **E** Utilization of ELISA to evaluate the binding affinity of galectin-3 to PGN, LTA, and WTA. **F** Determination of the MIC through microdilution assay to assess the effect of LTA and WTA on the inhibition of *S. suis* by galectin-3. **G** Zeta potential determination. **H** Percent of bacterial cell surface hydrophobicity. **I** Membrane potential was determined by fluorescence intensity measurements using a membrane potential probe, the DiOC_2_(3) (50 μM). Data are presented as mean ± SD derived from three independent experiments. One-way ANOVA was employed to evaluate statistical significance. NS, not significant; *P < 0.05; **P < 0.01; ***P < 0.001.

The above results demonstrate that galectin-3 could induce aggregation and bactericidal activity against *S. suis* in a β-galactoside-dependent manner. Therefore, we further explored which carbohydrates on the *S. suis* surface are bound by galectin-3. Peptidoglycan (PGN), lipoteichoic acid (LTA), and wall teichoic acid (WTA) were extracted from *S. suis*. As shown in Figure 6A, the PGN extracted from *S. suis* exhibited typical PGN absorption peaks in the 400–4000 cm⁻^1^ range, which were similar to those of commercial *Staphylococcus aureus* PGN: the C–O–C glycosidic bond at 1064 cm⁻^1^ may represent the NAG-(β-1,4)-NAM structure in the sample; the N–H bending vibrations coupled to C–N stretching vibrations of amide II occurred at approximately 1533 cm⁻^1^; the peak at 1676 cm⁻^1^ corresponded to C = O (the carbonyl stretching vibration); and the broad absorption band around 3282 cm⁻^1^ belonged to OH stretching absorption bands in the PGN. Figure 6B presented the Fourier transform infrared (FTIR) spectrum of LTA obtained from *S. suis* and that from commercial *Staphylococcus aureus*: a P-O stretching vibration peak was observed around 1038 cm⁻^1^, and the -COO^−^ absorption peak of alanine was observed at 1378 cm⁻^1^; vibration peaks at 1559 cm⁻^1^ and 1654 cm⁻^1^ corresponded to C = O and -NH bonds in α-D-N-acetylglucosamine; a peak around 2928 cm⁻^1^ was attributed to the -CH bond vibrations in the fatty acid chains; and the peak around 3424 cm⁻^1^ corresponded to -OH stretching vibrations. Figure 6C shows the WTA extracted from *S. suis*: a C–O–C stretching vibration absorption peak appeared around 1045 cm⁻^1^; the P-O bond (phosphorus-oxygen single bond) stretching vibration in the phosphate group was observed around 1103 cm⁻^1^; the characteristic absorption peak of the P = O phosphate group was observed around 1275 cm⁻^1^; and the -OH stretching vibration was observed around 3315 cm⁻^1^. Furthermore, lysozyme may effectively hydrolyze the PGN in the cell wall, breaking down the insoluble PGN into soluble glycopeptides, which can indirectly show the presence of the PGN. As shown in Figure 6D, lysozyme dissolution tests were performed on PGN extracted from *S. suis*, and the absorbance of the solution gradually decreased over the hydrolysis time, reaching a plateau after 7 h. This indicated that the extract can be completely digested and degraded by lysozyme, confirming that the extract was PGN. Results from the ELISA binding assay (Figure 6E) indicate that galectin-3 exhibited binding affinity for both LTA and WTA, with a stronger affinity for WTA. In MIC testing, the addition of LTA and WTA extracted from *S. suis* to galectin-3 resulted in a fourfold and 16-fold increase in MIC, respectively (Figure 6F). Following the binding of galectin-3 to the teichoic acids on the bacterial surface, alterations in the surface characteristics of the bacteria were observed. Specifically, the addition of galectin-3 resulted in an increase in the positive charge on the bacterial surface (Figure 6G), enhanced hydrophobicity (Figure 6H), and depolarization of the bacterial membrane potential (Figure 6I), thereby disrupting membrane homeostasis.

### Galectin-3 induces cell wall stress and disrupts protein synthesis in* S. suis*

**Figure 7:**
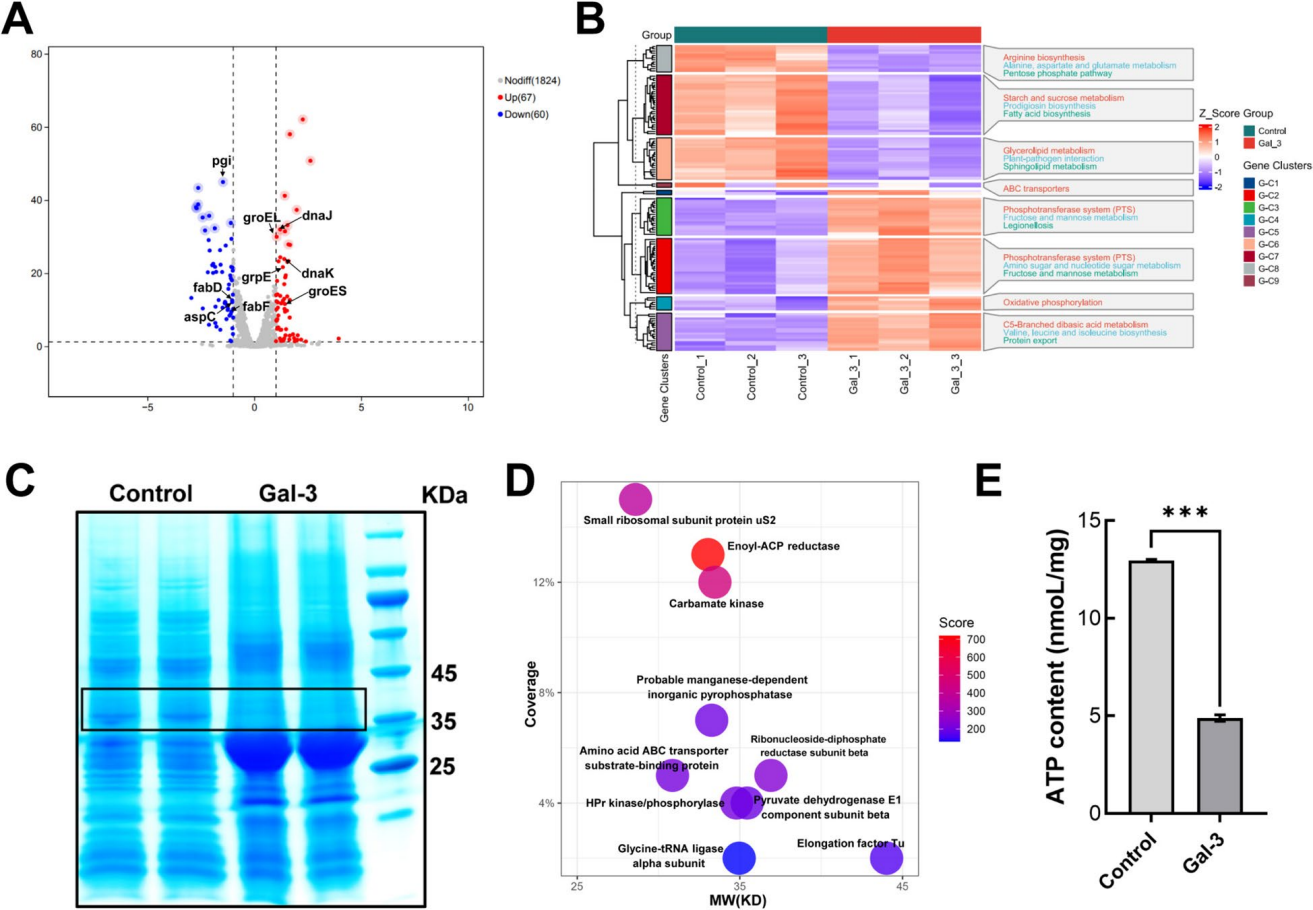
**Galectin-3 induces cell wall stress and disrupts protein synthesis in *S. suis.* A** Volcano map of differentially expressed genes (DEGs) in *S. suis* following 2 h of galectin-3 treatment (|log_2_FC|≥ 1 and false discovery rate [FDR] < 0.05). **B** KEGG enrichment analysis of DEG.** C** The SDS-PAGE gel electrophoresis of *S. suis* treated with galectin-3 for 2 h. **D** Nano LC–ESI–MS/MS analysis of the protein composition at 35 kDa in the SDS-PAGE gel electrophoresis of *S. suis* treated with galectin-3 for 2 h. **E** Intracellular ATP content was measured using an enhanced ATP assay kit following 2 h treatment of *S. suis* with galectin-3. One-way ANOVA was utilized to assess statistical significance. NS, not significant; *P < 0.05; **P < 0.01; *** *P* < 0.001.

To investigate the downstream effects of Galectin-3-mediated recognition of *S. suis*, we performed transcriptomic and proteomic analyses following bacterial exposure to galectin-3. Transcriptome sequencing revealed that galectin-3 stimulation upregulated bacterial cell wall stress response pathways, including genes associated with cell wall repair, molecular chaperone expression, and protein folding, such as dnaK, dnaJ, groEL, groES, and grpE (Figure 7A). The results of Kyoto Encyclopedia of Genes and Genomes (KEGG) gene annotation and enrichment analysis show that the differentially expressed genes (DEG) were mostly related to core metabolic pathways and responses to cell wall stress (Figure 7B). We further examined the protein profile of *S. suis* following galectin-3 treatment. Notably, compared with the untreated control group, SDS-PAGE analysis revealed the disappearance of a distinct protein band around 35 kDa in the galectin-3-treated group (Figure 7C). The band corresponding to 35 kDa from the control group was excised and subsequently analyzed using Nano-LC–ESI–MS/MS, with the results presented in Figure 7D. Detailed raw data from the nano-LC–ESI–MS/MS analysis presented in Figure 7D are available in Additional file 5. The main components of this band were recognized as enoyl-ACP reductase (FabK), carbamate kinase (CK), and small ribosomal subunit protein uS2 (rpsB). Furthermore, RT-qPCR validated that the mRNA expression levels of these three proteins were also notably reduced after galectin-3 treatment (Additional file 6A). Furthermore, molecular docking predictions (Additional file 6B) and pull-down assay (Additional files 6C and 6D) results demonstrate that galectin-3 directly interacts with FabK and rpsB. FabK, CK, and rpsB play crucial roles in essential cellular functions, including the synthesis of fatty acids, energy metabolism, and protein synthesis. These processes are interconnected through the production and use of ATP during the growth, proliferation, and adaptation of bacteria. Figure 7E illustrates that ATP synthesis in *S. suis* was markedly diminished after the administration of galectin-3. To investigate whether galectin-3 directly binds to bacterial genomic DNA or induces DNA damage to inhibit bacterial proliferation, UV–visible absorption spectrum and gel retardation assays were conducted. Additional file 7A illustrates that the incorporation of galectin-3 could not induce the shift of the peak absorption intensity of the genome DNA of bacteria. Additionally, as illustrated in Additional file 7B, galectin-3 did not inhibit the migration of bacterial genomic DNA in the agarose gel electrophoresis. These results indicate that galectin-3 inhibited protein synthesis in *S. suis* but did not interact with bacterial genomic DNA.

### Intranasal administration of galectin-3 accelerates the clearance of *S. suis* in mice

**Figure 8:**
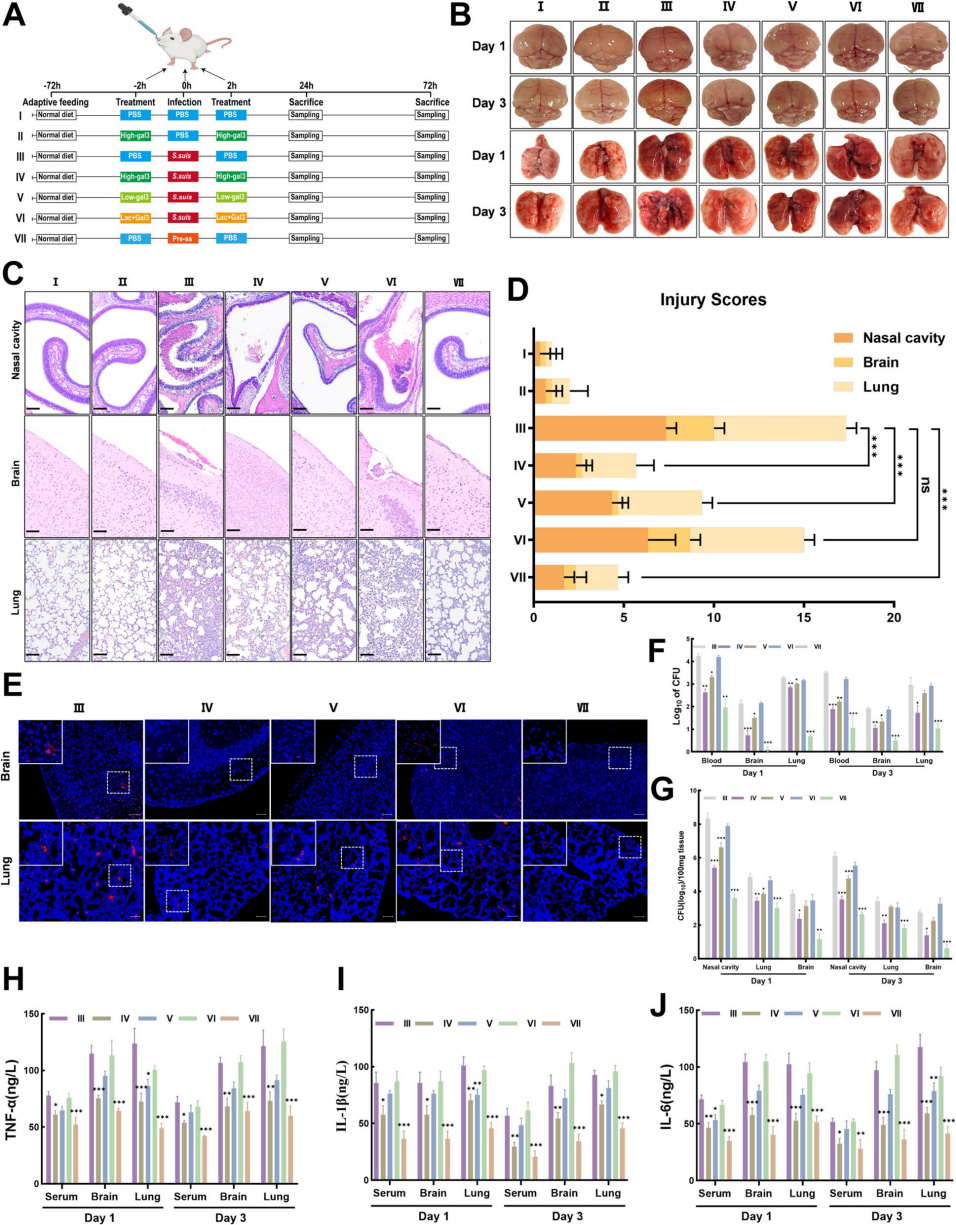
**Intranasal administration of galectin-3 accelerates the clearance of *S. suis *in the mouse infection model. A** The schematic representation of the in vivo antibacterial assay utilizing galectin-3. A total of 84 mice were divided into seven groups: a blank control group (I), a galectin-3 control group (II), an *S. suis* infection group (III), a high-dose galectin-3 protection group (4 mg/kg) (IV), a low-dose galectin-3 protection group (2 mg/kg) (V), a lactose and galectin-3 co-incubation group (VI), and an *S. suis* pre-incubated with galectin-3 group (VII). Mice were infected intranasally with 2 × 10⁹ CFU of *S. suis*. The blank control and galectin-3 control groups were administered PBS intranasally. (Schematic of the mouse model derived from BioRender) **B** Representative images of intact brains and lungs from each group of mice 1 day and 3 days post-infection. **C-D** Representative hematoxylin and eosin (HE) staining images and pathological damage scores of nasal cavities, brains, and lungs from each group of mice 1 day post-infection. **E** In situ hybridization was performed to detect the bacterial load of *S. suis* in the nasal cavities, brains, and lungs of mice in each group. **F** Colony counts were determined using blood and tissue homogenates (brains and lungs) collected 1 day and 3 days post-infection. **G** Bacterial loads in the nasal cavities, brains, and lungs of mice were quantified using reverse transcription-quantitative polymerase chain reaction (RT-qPCR) based on the standard curve of *S. suis* (left panel). **H-J** Levels of TNF-α, IL-1β, and IL-6 in serum, brain, and lung tissues were quantified using ELISA. Data are expressed as mean ± SD from three independent experiments. One-way ANOVA was utilized to assess statistical significance. NS, not significant; *P < 0.05; **P < 0.01; ***P < 0.001.

We developed a nasal infection model of *S. suis* in BALB/c mice to examine the antibacterial activity of galectin-3. *S. suis* was identified in the nasal cavity, blood, lungs, and brain at 1 day and 3 days post-infection. Necropsy of the brain and lungs revealed visible congestion and hemorrhage in mice from both the *S. suis* infection group (III) and the lactose combined galectin-3 co-incubation group (VI) (Figure 8B). Histological analysis with H&E staining of nasal, brain, and lung tissue sections revealed pathological damage in the *S. suis* infection group and the lactose combined galectin-3 co-incubation group (Figure 8C and D). Irregular arrangement of mucosal epithelial cells and glands, scattered necrosis, nuclear fragmentation, and infiltration of neutrophils and lymphocytes were observed, along with small focal hemorrhages. Necrotic debris and neutrophils were present in the nasal cavity, with focal hemorrhages observed in the meninges. The lungs displayed extensive thickening of alveolar walls, widened alveolar septa, narrowed alveolar spaces, and irregularly shaped bronchioles. Additionally, small foci of lymphocyte infiltration and sporadic perivascular hemorrhages were noted. Intranasal administration of galectin-3 significantly reduced these pathological damages, with the most significant effects observed in the high-dose galectin-3 group (IV) and the galectin-3 combined *S. suis* pre-incubation group (VII). Additionally, results from in situ hybridization (Figure 8E), tissue homogenate colony plating counts (Figure 8F), and RT-qPCR (Figure 8G) demonstrate that the administration of galectin-3 significantly decreased bacterial loads in the blood, nasal cavity, brain, and lung tissues. Furthermore, as illustrated in (Figure 8H-J), intranasal galectin-3 administration reduced the upregulation of inflammatory cytokines induced by *S. suis* infection. These results demonstrate that galectin-3 could accelerate the clearance of *S. suis* in mice.

## Discussion

The nasal cavity, as the entry point of the respiratory tract, is continuously exposed to the outside world, making it susceptible to pathogen infiltration through the nasal mucosa. The nasal mucus layer covering the nasal mucosa serves as the first physical and chemical barrier against the invasion of pathogens [24, 25]. Current research on antimicrobial proteins in nasal mucus has predominantly concentrated on a few proteins, including mucins, lactoferrin, lysozyme, and defensins. Nonetheless, it remains unknown whether the existence of additional antimicrobial proteins in nasal mucus and their respective mechanisms of action are still largely unexplored [26]. Due to the similar anatomy and tissue structure of the nasal cavity of goats, they resemble those of humans [27, 28]. In addition, the goat nasal mucosal explant model employed in this study maintained good activity in vitro, making it an ideal model to investigate the composition and functions of nasal mucus [29]. In this study, total nasal mucus proteins were collected from the goat nasal mucosal explant model. The components under 30 kDa demonstrated the highest antimicrobial activity, and we found that galectin-3 was an antimicrobial protein from this fraction.

Galectin-3 is a chimera-type galectin with a C-terminal carbohydrate-recognition-binding domain and an N-terminal proline-and-glycine-rich domain. Galectin-3 is a member of an evolutionarily conserved protein family and is found in the nucleus, cytoplasm, cell membrane, or is secreted extracellularly [30–32]. Galectins bind to glycosylated structures on cell surfaces through β-galactoside, initiating transmembrane signaling cascades that govern multiple biological processes such as tissue repair and regeneration, immune cell activation, apoptosis, cell migration and adhesion, cytokine secretion, and tumorigenesis [33, 34]. Galectins can also bind to glycosylated structures on the surface of pathogenic microorganisms and play a role in the infection process [35, 36]. In an experimental infection with *Cryptococcus neoformans*, the absence of galectin-3 resulted in an increased bacterial load in the organs of mice and contributed to a skewed TH17 immune response in the host. Additionally, recombinant galectin-3 interacted with the capsule of the H99 strain, effectively inhibiting its growth in vivo [37]. Galectin-3 was selectively expressed by the gastric surface epithelial cells and exhibited aggregation and bactericidal activity against *Helicobacter pylori* in a β-galactoside-dependent manner [38]. Galectin-4 and galectin-8 also bind to certain *Escherichia coli* strains expressing human blood group antigens via their CRD domains and subsequently eliminate them [39]. In our study, galectin-3 was initially identified in nasal mucus, primarily produced by mucosal epithelial cells and submucosal glands, with secretion levels rising with age. Furthermore, intranasal infection with *S. suis* induced an increase in galectin-3 expression, indicating a potential role for galectin-3 in the response to bacterial infections. Recombinant galectin-3 was obtained and shown to bind to bacterial teichoic acids in a β-galactoside-dependent manner, resulting in bacterial aggregation and subsequent elimination of *S. suis*. In a mouse model of intranasal infection with *S. suis*, intranasal administration of galectin-3 decreased bacterial load in multiple organs and alleviated the pathological damage resulting from the infection. Our findings suggest that galectin-3 has the potential as an antimicrobial agent for future biomedical applications.

Although the binding of galectin-3 to bacterial cell wall components has been widely validated, the mechanisms underlying its direct antibacterial effects are seldom reported. The isoelectric point of galectin-3 is 9.19, suggesting it possesses a positive charge at a physiological pH. This facilitates the formation of robust electrostatic attractions with negatively charged molecules on bacterial surfaces, potentially disrupting bacterial membrane integrity [40]. For instance, the C-type lectin RegIIIα, secreted by intestinal epithelial cells, recognizes Gram-positive bacteria by binding to peptidoglycan carbohydrates and kills bacteria by forming a hexameric membrane-penetrating pore [41]. In a similar manner, small proline-rich protein 2A (SPRR2A) is activated in response to bacterial infections occurring in the stomach, lungs, and skin. Electrostatic interactions facilitate the association of SPRR2A with bacterial membranes, leading to the selective destruction of Gram-positive bacteria through the disruption of their cell membranes [42]. Similar mechanisms by which galectin-3 attached to *S. suis* cell walls, result in an increase in the surface electrostatic charge of the bacteria. As galectin-3 accumulated on the bacterial surface, its hydrophobic amino acid residues inserted into the bilayer structure of the bacterial membrane, altering the membrane domain organization. This increased the hydrophobicity of the membrane surface, depolarized the membrane potential, and changed membrane permeability, ultimately leading to bacterial lysis.

In numerous bacteria, exposure to antimicrobials that target cell wall synthesis leads to significant changes in protein production, indicating a complex adaptive response to cell wall stress [43]. For example, LL-37, the only human cathelicidin-derived antimicrobial peptide, exhibits bactericidal activity primarily through membrane-targeting and intracellular mechanisms. The multimeric N-terminal domain interacts with bacterial phospholipid bilayers, disrupting the ordered arrangement of membrane phospholipids, increasing membrane permeability, and compromising membrane integrity. This results in the uncontrolled efflux of intracellular contents and ultimately leads to cell lysis [44]. Beyond its membrane-disruptive action, LL-37 is also capable of penetrating bacterial cells, where it inhibits nucleic acid and protein synthesis, impairs enzymatic activities, and interferes with cell wall biosynthesis [45]. Collectively, these multifaceted mechanisms synergistically disrupt essential cellular functions, culminating in bacterial death. In this study, transcriptomic analysis of *S. suis* exposed to galectin-3 revealed significant suppression of several metabolic pathways. Notably, arginine biosynthesis and alanine, aspartate, and glutamate metabolism were significantly downregulated, indicating a limited supply of precursors essential for protein synthesis, which likely disrupts the production of structural proteins and enzymes. In addition, the repression of the pentose phosphate pathway suggests impaired generation of reducing equivalents (e.g., NADPH) and nucleotide precursors, thereby blocking nucleic acid synthesis and antioxidant defence. Downregulation of fatty acid biosynthesis, glycerolipid metabolism, and sphingolipid metabolism indicates disrupted membrane biogenesis and integrity, providing molecular evidence of membrane damage induced by galectin-3. Additionally, suppression of ABC transporter systems, which mediate the influx of nutrients and efflux of toxins, indicates diminished environmental adaptability and stress resistance. The decreased expression of genes involved in prodigiosin biosynthesis and plant-pathogen interaction pathways may reflect attenuation of virulence-associated functions. Conversely, the upregulation of the phosphotransferase system (PTS), fructose and mannose metabolism, and amino sugar and nucleotide sugar metabolism pathways suggests a compensatory response aimed at enhancing sugar uptake and utilization under carbon and energy limitation. Enhanced oxidative phosphorylation indicates an elevated energy demand under stress conditions. Meanwhile, the induction of valine, leucine, and isoleucine biosynthesis and protein export pathways may represent a reactive compensation for cellular damage, albeit at the cost of intensified metabolic burden, potentially accelerating cellular collapse. The analysis of the proteome of *S. suis* treated with galectin-3 using Nano LC–ESI–MS/MS confirmed the downregulation of three essential proteins: enoyl-ACP reductase (FabK), carbamate kinase (CK), and small ribosomal subunit protein uS2 (rpsB). All three proteins, FabK, CK, and rpsB, are dependent on energy molecules such as ATP and NADH in bacterial metabolism. In alignment with the proteomic findings, the addition of galectin-3 led to a decrease in bacterial ATP levels and a reduction in the NAD^+^ /NADH ratio. FabK facilitates the last step in the bacterial fatty acid elongation cycle within the process of de novo fatty acid synthesis, predominantly converting enoyl-ACP to acyl-ACP, thereby promoting the elongation of fatty acid chains. This reaction employs NADH or NADPH as electron donors, supplying the essential reducing power [46]. The pathway of fatty acid synthesis equips bacteria with crucial lipid molecules that play a significant role in membrane formation and energy storage, making ACP reductase a potential target for antimicrobial drugs, especially in the context of growing antibiotic resistance [47, 48]. CK is an enzyme involved in nitrogen metabolism, catalyzing the conversion of carbamoyl phosphate to carbamate, generating ATP in the process. Carbamate serves as a crucial intermediate in the arginine biosynthesis pathway, where arginine plays a vital role as an essential amino acid for bacterial protein synthesis and overall cellular function [49, 50]. RpsB is a core protein of the bacterial small ribosomal subunit (30S), contributing to ribosome assembly, the initiation of translation, and the maintenance of both accuracy and efficiency during the translation process [51]. Taken together, galectin-3 disrupts core metabolic and biosynthetic pathways, compromises membrane and cell wall integrity, suppresses virulence factor expression, and induces compensatory upregulation for the stress response. Nonetheless, the exact molecular mechanisms through which galectin-3 suppressed the expression of FabK, CK, and rpsB, as well as the regulatory interactions between these key molecules, require further investigation.

**Figure 9:**
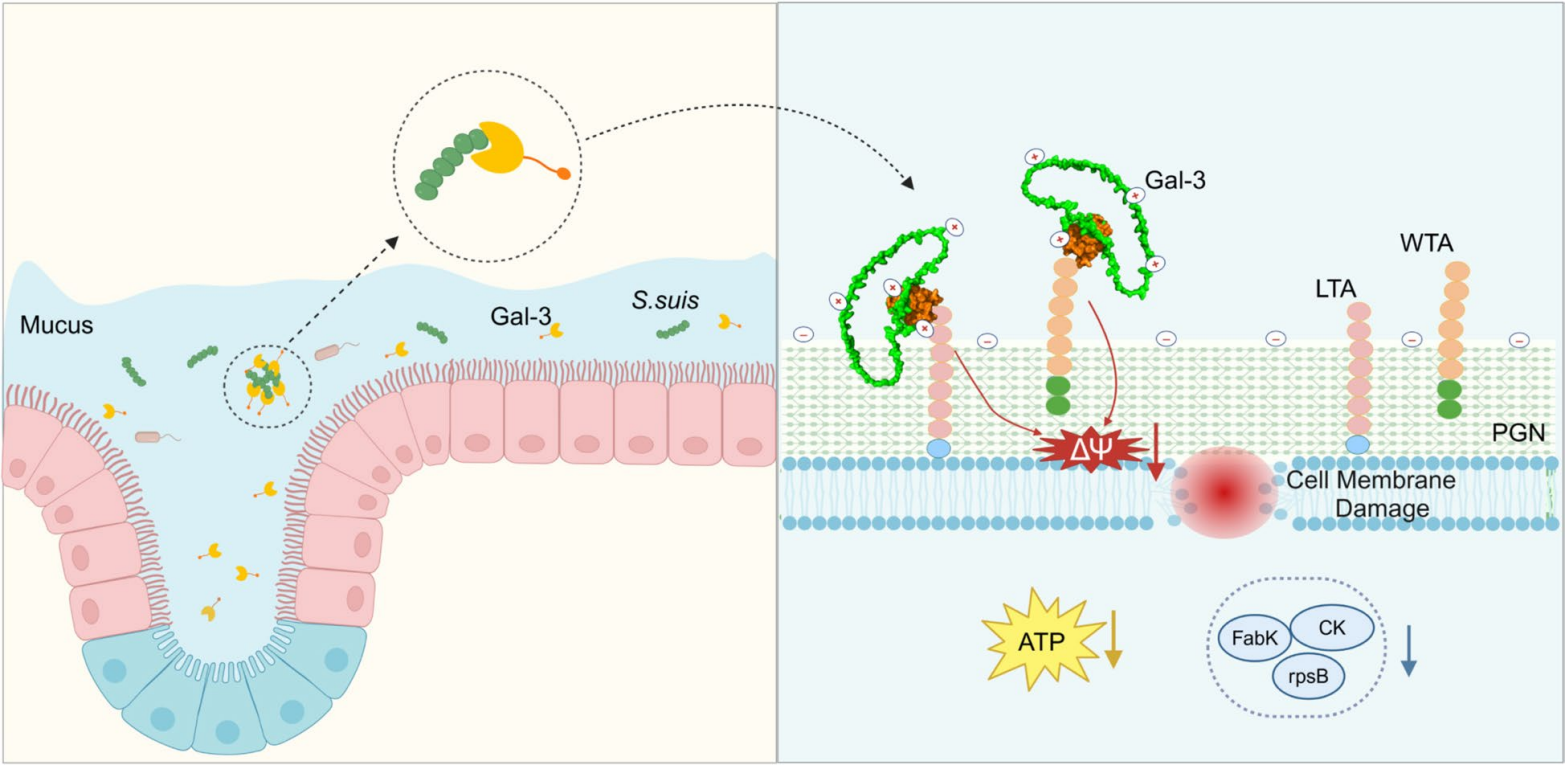
**Protective mechanism of galectin-3 against *S. suis* infection in the nasal mucus. **Schematic diagram of the mechanism by which nasal galectin-3, a protein found in nasal mucus, resists *S. suis* infections (Created in BioRender). Galectin-3 binds to *S. suis* in a teichoic acid-dependent manner, increasing the number of positive charges on the bacterial membrane, enhancing surface hydrophobicity, and causing membrane depolarization, thereby disrupting membrane integrity. Additionally, galectin-3 inhibits the synthesis of bacterial proteins FabK, CK, and rpsB, further suppressing the growth and metabolism of *S. suis*, ultimately exerting bactericidal effects.

In summary, our study is the first to identify the presence of galectin-3 in the nasal mucus and demonstrates its antibacterial activity against *S. suis*. As illustrated in Figure 9, galectin-3 bound to the surface glycans of *S. suis* in a β-galactoside-dependent manner. The aggregation of galectin-3 on the bacterial membrane neutralized the negative surface charge of the bacteria, increased surface hydrophobicity, and induced membrane depolarization, ultimately compromising bacterial integrity. Additionally, galectin-3 suppressed the expression of FabK, CK, and rpsB within *S. suis*, further disrupting bacterial growth and metabolism. These findings highlighted the potential role of galectin-3 as a host defense factor, offering fresh perspectives for the advancement of antimicrobial therapeutic strategies.

## Supplementary Information

**Additional file 1.** **Summary of identified nasal mucus proteins with molecular weight less than 30 kDa.**

**Additional file 2. Prokaryotic expression of His-galectin-3, GST-galectin, His-CK, His-FabK, and His-rpsB. A **Schematic map of the recombinant expression plasmid pET22b-galectin-3. **B** pET22b-galectin-3 plasmid was identified by BamHI digestion, as well as BamHI and NdeI dual digestion, and subsequently confirmed via agarose gel electrophoresis. **C **The recombinant plasmid pET22b-galectin-3 was transformed into *Escherichia coli* strain BL21. 0.2 mM isopropyl β-d-thiogalactopyranoside was added to induce protein expression when the OD_600_ of cells was about 0.5. SDS-PAGE analysis of galectin-3 expression in *Escherichia coli* after Coomassie Brilliant Blue staining: lane 1: induced supernatant from the empty vector control; lane 2: inclusion bodies from the empty vector control; lane 3: non-induced supernatant from the pET22b-galectin-3; lane 4: inclusion bodies from the non-induced pET22b-galectin-3; lane 5: induced supernatant from the pET22b-galectin-3; lane 6: inclusion bodies from the induced pET22b-galectin-3; lane 7: the purified protein His-galectin-3. **D** Western blot validation of purified galectin-3. Lane 1: supernatant from induced recombinant strains following sonication, prior to purification using the Ni-NTA column; Lane 2: flow-through; Lane 3: wash fraction; Lane 4: elution fraction; Lane 5: purified recombinant galectin-3 post-desalting. **E-H** Plasmid maps of pGEX-4T-galectin3, pET22b-CK, pET22b-FabK, and pET22b-rpsB, respectively. **I-L** Recombinant plasmids pGEX-4T-galectin3, pET22b-CK, pET22b-FabK, and pET22b-rpsB were identified by single digestion with BamHI and double digestion with BamHI/EcoRI, while pET22b-rpsB was identified by digestion with BamHI and NotI.** M-P** Analysis of protein expression for GST-galectin-3, His-CK, His-FabK, and His-rpsB using SDS-PAGE. Lane 1: supernatant from induced empty vector control; lane 2: inclusion bodies from the induced empty vector; lane 3: non-induced supernatant from the pGEX-4T-galectin3, pET22b-CK, pET22b-FabK, and pET22b-rpsB, respectively; lane 4: inclusion bodies from the non-induced recombinant plasmids; lane 5: induced supernatant from the pGEX-4T-galectin3, pET22b-CK, pET22b-FabK, and pET22b-rpsB, respectively; lane 6: inclusion bodies from the induced recombinant plasmids; lane 7: purified protein GST-galectin-3.

**Additional file 3. SEM and TEM were employed to evaluate the impact of lactose on the ultrastructural damage to bacteria caused by galectin-3.**

**Additional file 4. The CRD domains of galectin-3 in goats, pigs, and humans demonstrate a highly conserved structure and function. A **Amino acid sequence alignment of galectin-3 in goats, pigs, and humans. **B** Structural similarity investigation of galectin-3 in goats, pigs, and humans using PyMOL. (a-c) Structures of the galectin-3 CRD from goats, pigs, and humans, respectively. (d-f) Structural alignments of galectin-3 CRD between goats and pigs, goats and humans, and humans and pigs, respectively. **C **Immunohistochemical staining was used to detect the distribution of galectin-3 in the vestibular, respiratory, and olfactory areas of the nasal cavity in 60-day-old pigs. **D **Statistical comparisons of galectin-3 immunohistochemical staining in 60-day-old pigs and goats. **E **Galectin-3 levels in homogenized nasal mucosa samples from various regions of 60-day-old goats and pigs were analysed using ELISA. **F-G **Determination of the MIC of porcine and human galectin-3 against *S. suis* using the microdilution method. Data are expressed as mean ± SD from three independent experiments. One-way ANOVA was utilized to assess statistical significance. NS, not significant; *P < 0.05; **P < 0.01; ***P < 0.001.

**Additional file 5. Summary of proteins identified after galectin-3 treatment of *S. suis.***

**Additional file 6. Galectin-3 interacts with FabK and rpsB. A **RT-qPCR analysis of mRNA expression levels of FabK, CK, and rpsB in *S. suis* treated with galectin-3 for 2 h. **B **Molecular docking predictions of galectin-3 interactions with FabK (a), CK (b), and rpsB (c), respectively. **C** Ni-NTA magnetic beads were used to enrich streptococcal cell-associated proteins interacting with His-galectin-3, followed by identification of FabK and rpsB through nano-LC-ESI-MS/MS analysis. The secondary mass spectrum of FabK and rpsB confirms their specific binding to His-galectin-3. **D** GST pull-down assays were performed using GST-galectin-3. His-CK, His-FabK, and His-rpsB were incubated with GST or GST-galectin-3 immobilized on glutathione-sepharose beads. Bound proteins were analyzed by SDS-PAGE and detected by western blotting.

**Additional file 7. Galectin-3 does not interact with bacterial genomic DNA. A** UV spectrum analysis of galectin-3 co-incubated with *S. suis* genomic DNA at different concentrations for 1 h, measured within the 200-400 nm wavelength range. **B** Agarose gel electrophoresis (0.7%) showing the effect of galectin-3 at different concentrations on the migration of *S. suis* genomic DNA after 1 h of co-incubation.

**Additional file 8. Replicates of bacterial colony assays. A-C **Representative images of bacterial colonies on agar plates from three different experiments of Figure 2B. **D-E** Two additional independent experiments of bacterial colonies on agar plates from three different experiments of Figure 3A.

**Additional file 9. SDS-PAGE of protein expression replicates. **The SDS-PAGE results from the two additional independent experiments of protein expression for His-galectin-3 (A and B), GST-galectin-3 (C and D), His-CK (E and F), His-FabK (G and H), and His-rpsB (I and J), respectively.

**Additional file 10. The total ion chromatogram (TIC) profile of tryptic digestion peptides. **The TIC shows the mass spectrometry peak profile of the sample following tryptic digestion, with annotated peaks corresponding to the identified peptides in Figure 7C.

**Additional file 11. Visual evidence of bacterial aggregation. A** Images showing bacterial aggregation and sedimentation in test tubes following galectin-3 treatment. **B** Images demonstrating that lactose addition inhibited galectin-3-induced aggregation and sedimentation of *S. suis*.

## Data Availability

The data that support the findings of this study are available from the corresponding author upon reasonable request.
