# Supplementary material for: Galectin-3: a novel antimicrobial host factor identified in goat nasal mucus
Source: Vet Res. 2025 Jul 21;56:153. doi: 10.1186/s13567-025-01586-w (PMC12281822; doi:10.1186/s13567-025-01586-w)
Supplement: Supplementary file 1 — Additional file 1. Summary of identified nasal mucus proteins with molecular weight less than 30 kDa. [file 13567_2025_1586_MOESM1_ESM.docx]

**Additional file 1. Summary of identified nasal mucus proteins with molecular weight less than 30 kDa**

| Protein FDR Confidence: Combined | Accession | Description | Exp. q-value: Combined | Sum PEP Score | Coverage [%] | #Peptides | #PSMs | #Unique Peptides | #AAs | MW[kDa] | calc. pI | Score Sequest HT: Sequest HT | #Peptides (by Search Engine): Sequest HT | #Razor Peptides | Abundances (Scaled) | Found in Sample |
| --- | --- | --- | --- | --- | --- | --- | --- | --- | --- | --- | --- | --- | --- | --- | --- | --- |
| High | A0A452EUK4 | Inorganic diphosphatase OS=Capra hircus OX=9925 GN=PPA1 PE=3 SV=1 | 0 | 50.608 | 56 | 9 | 12 | 4 | 289 | 32.8 | 5.47 | 30.55 | 9 | 3 | 100 | High |
| High | P00712 | Alpha-lactalbumin OS=Capra hircus OX=9925 GN=LALBA PE=1 SV=1 | 0 | 53.94 | 50 | 8 | 15 | 8 | 142 | 16.2 | 5.3 | 33.68 | 8 | 0 | 100 | High |
| High | A0A452G2G1 | Protein S100 OS=Capra hircus OX=9925 GN=S100A4 PE=3 SV=1 | 0 | 25.46 | 44 | 6 | 12 | 6 | 101 | 11.8 | 6.11 | 17.05 | 6 | 0 | 100 | High |
| High | A0A452EEM4 | Peptidoglycan-recognition protein OS=Capra hircus OX=9925 GN=PGLYRP1 PE=3 SV=1 | 0 | 45.808 | 45 | 6 | 8 | 6 | 190 | 21 | 9.14 | 28.21 | 6 | 0 | 100 | High |
| High | P82018 | Cathelicidin-2 OS=Capra hircus OX=9925 GN=CATHL2 PE=1 SV=1 | 0 | 12.65 | 37 | 4 | 7 | 3 | 176 | 19.8 | 9.16 | 16.01 | 4 | 0 | 100 | High |
| High | A0A452DJV2 | Secretoglobin family 1C member 1 OS=Capra hircus OX=9925 GN=SCGB1C1 PE=3 SV=1 | 0 | 10.965 | 27 | 3 | 6 | 3 | 95 | 10.1 | 4.75 | 13.1 | 3 | 0 | 100 | High |
| High | Q0PGY0 | Lingual antimicrobial peptide OS=Capra hircus OX=9925 GN=LAP PE=2 SV=1 | 0 | 7.86 | 24 | 2 | 6 | 2 | 82 | 9.4 | 11.87 | 7.05 | 2 | 0 | 100 | High |
| High | A0A8C2P1Y7 | Galectin 3 OS=Capra hircus OX=9925 PE=4 SV=1 | 0 | 24.349 | 18 | 4 | 9 | 4 | 267 | 28.3 | 9.19 | 10.18 | 4 | 0 | 100 | High |
| High | A0A8C2P0L5 | Cathepsin Z OS=Capra hircus OX=9925 PE=4 SV=1 | 0 | 36.841 | 19 | 5 | 13 | 5 | 318 | 35.8 | 6.37 | 18.99 | 5 | 0 | 100 | High |
| High | P02756 | Beta-lactoglobulin OS=Capra hircus OX=9925 GN=LGB PE=1 SV=2 | 0 | 4.315 | 8 | 1 | 5 | 1 | 180 | 20 | 5.64 | 8.45 | 1 | 0 | 100 | High |
