# Supplementary figures and images for: Galectin-3: a novel antimicrobial host factor identified in goat nasal mucus
Source: Vet Res. 2025 Jul 21;56:153. doi: 10.1186/s13567-025-01586-w (PMC12281822; doi:10.1186/s13567-025-01586-w)

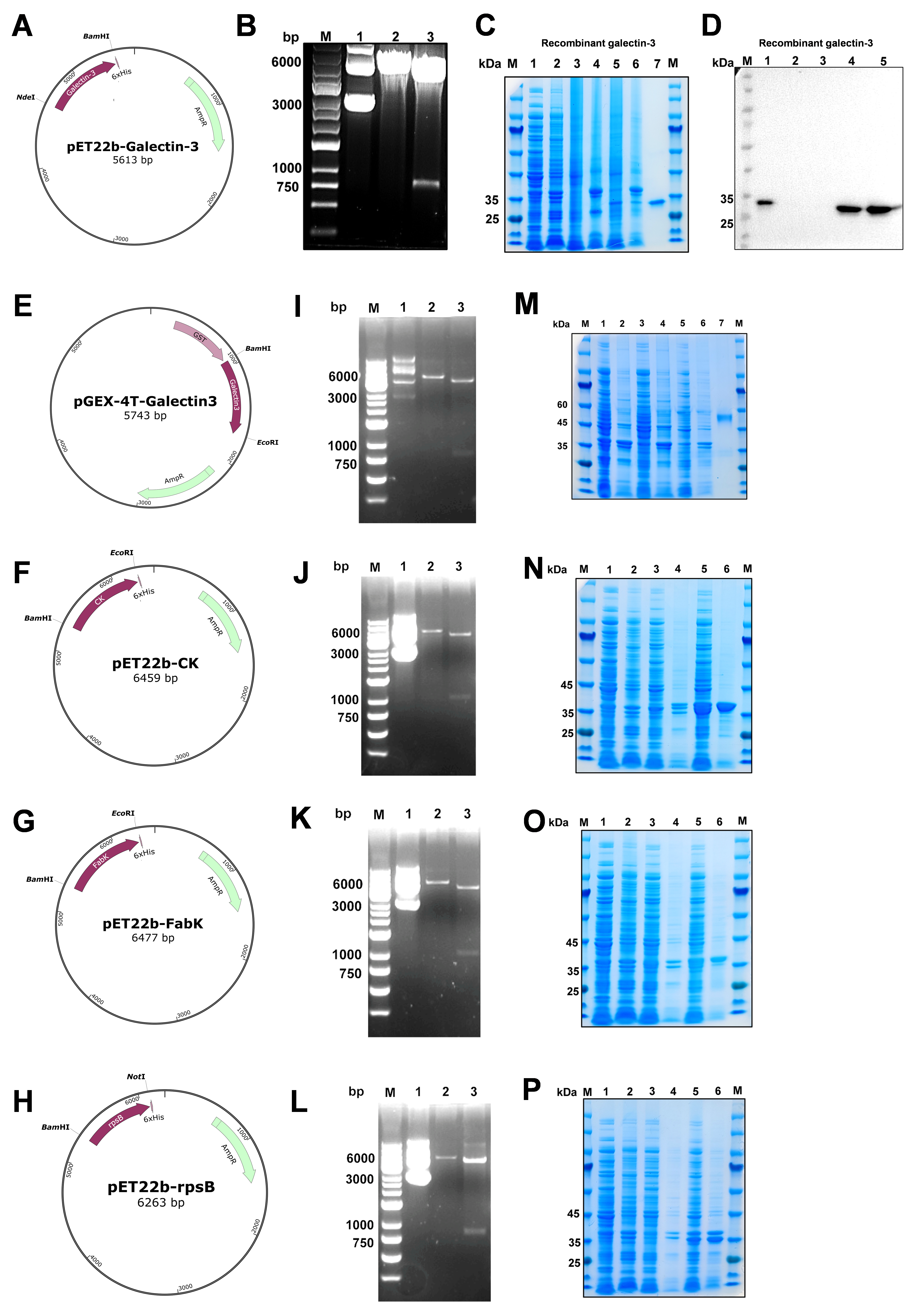

Supplement: Supplementary file 2 — Additional file 2. Prokaryotic expression of His-galectin-3, GST-galectin, His-CK, His-FabK, and His-rpsB. A Schematic map of the recombinant expression plasmid pET22b-galectin-3. B pET22b-galectin-3 plasmid was identified by BamHI digestion, as well as BamHI and NdeI dual digestion, and subsequently confirmed via agarose gel electrophoresis. C The recombinant plasmid pET22b-galectin-3 was transformed into Escherichia coli strain BL21. 0.2 mM isopropyl β-d-thiogalactopyranoside was added to induce protein expression when the OD600 of cells was about 0.5. SDS-PAGE analysis of galectin-3 expression in Escherichia coli after Coomassie Brilliant Blue staining: lane 1: induced supernatant from the empty vector control; lane 2: inclusion bodies from the empty vector control; lane 3: non-induced supernatant from the pET22b-galectin-3; lane 4: inclusion bodies from the non-induced pET22b-galectin-3; lane 5: induced supernatant from the pET22b-galectin-3; lane 6: inclusion bodies from the induced pET22b-galectin-3; lane 7: the purified protein His-galectin-3. D Western blot validation of purified galectin-3. Lane 1: supernatant from induced recombinant strains following sonication, prior to purification using the Ni-NTA column; Lane 2: flow-through; Lane 3: wash fraction; Lane 4: elution fraction; Lane 5: purified recombinant galectin-3 post-desalting. E-H Plasmid maps of pGEX-4T-galectin3, pET22b-CK, pET22b-FabK, and pET22b-rpsB, respectively. I-L Recombinant plasmids pGEX-4T-galectin3, pET22b-CK, pET22b-FabK, and pET22b-rpsB were identified by single digestion with BamHI and double digestion with BamHI/EcoRI, while pET22b-rpsB was identified by digestion with BamHI and NotI. M-P Analysis of protein expression for GST-galectin-3, His-CK, His-FabK, and His-rpsB using SDS-PAGE. Lane 1: supernatant from induced empty vector control; lane 2: inclusion bodies from the induced empty vector; lane 3: non-induced supernatant from the pGEX-4T-galectin3, pET22b-CK, pET22b-FabK, and pE [file 13567_2025_1586_MOESM2_ESM.tif]

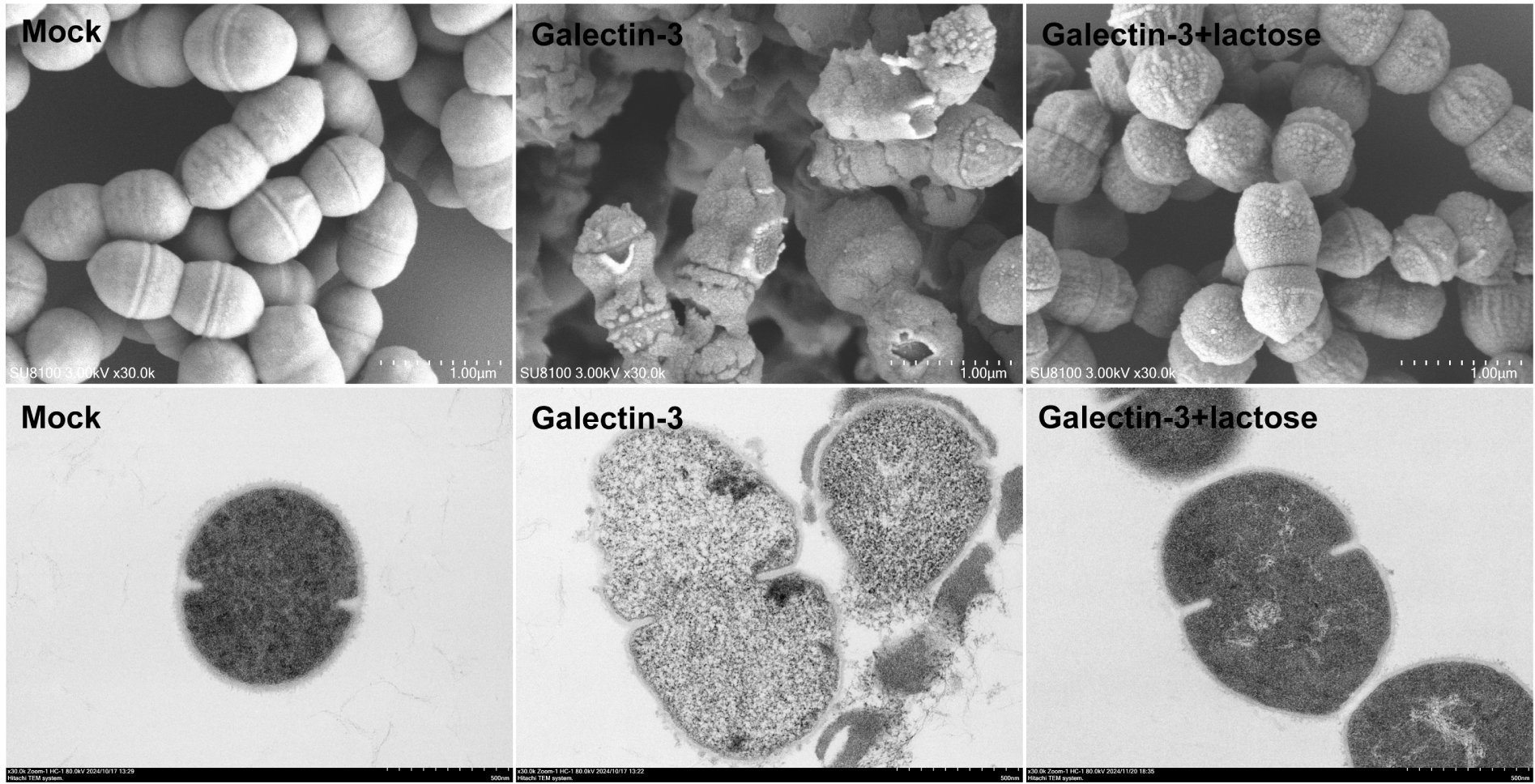

Supplement: Supplementary file 3 — Additional file 3. SEM and TEM were employed to evaluate the impact of lactose on the ultrastructural damage to bacteria caused by galectin-3. [file 13567_2025_1586_MOESM3_ESM.tif]

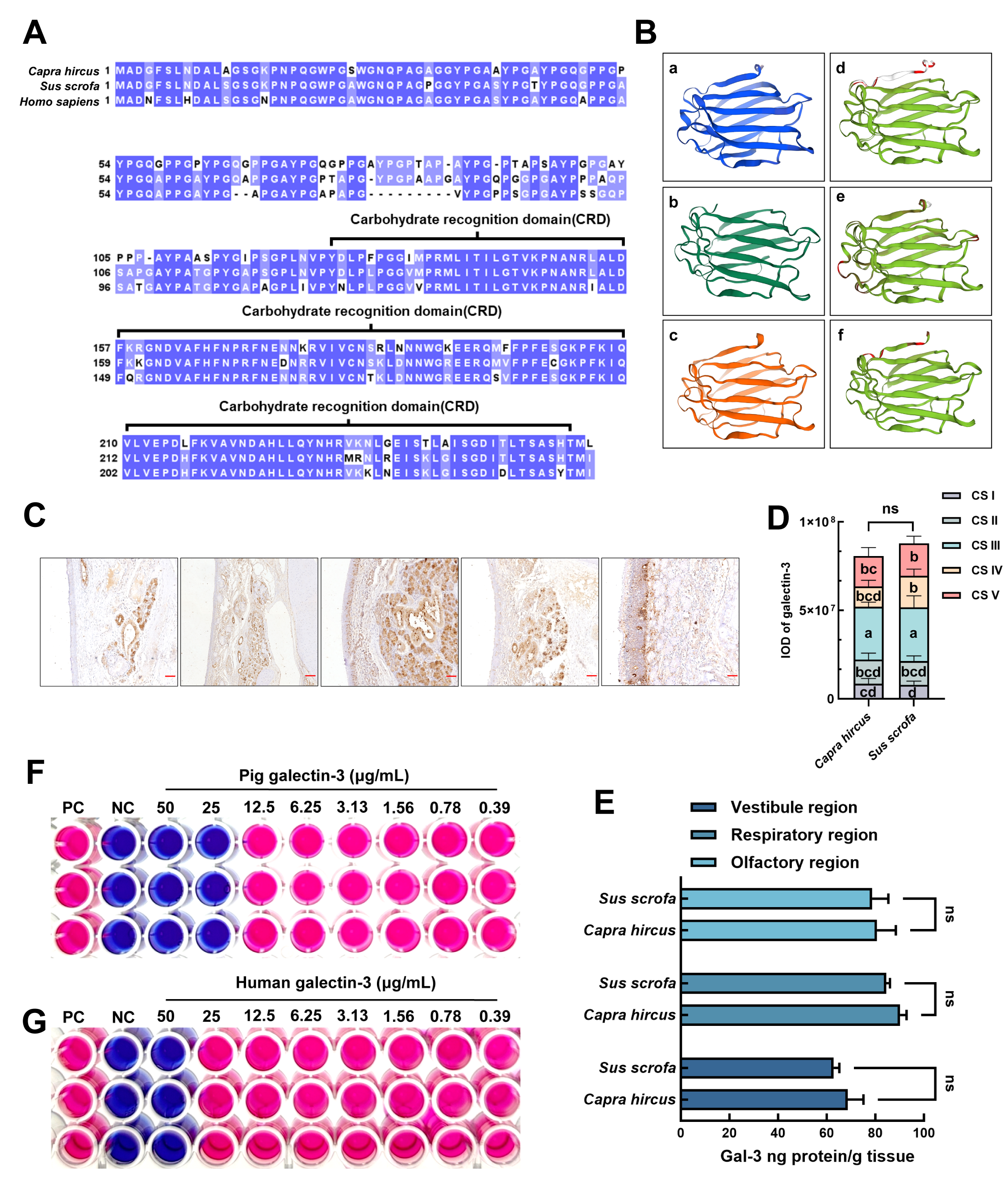

Supplement: Supplementary file 4 — Additional file 4. The CRD domains of galectin-3 in goats, pigs, and humans demonstrate a highly conserved structure and function. A Amino acid sequence alignment of galectin-3 in goats, pigs, and humans. B Structural similarity investigation of galectin-3 in goats, pigs, and humans using PyMOL. (a-c) Structures of the galectin-3 CRD from goats, pigs, and humans, respectively. (d-f) Structural alignments of galectin-3 CRD between goats and pigs, goats and humans, and humans and pigs, respectively. C Immunohistochemical staining was used to detect the distribution of galectin-3 in the vestibular, respiratory, and olfactory areas of the nasal cavity in 60-day-old pigs. D Statistical comparisons of galectin-3 immunohistochemical staining in 60-day-old pigs and goats. E Galectin-3 levels in homogenized nasal mucosa samples from various regions of 60-day-old goats and pigs were analysed using ELISA. F-G Determination of the MIC of porcine and human galectin-3 against S. suis using the microdilution method. Data are expressed as mean ± SD from three independent experiments. One-way ANOVA was utilized to assess statistical significance. NS, not significant; *P < 0.05; **P < 0.01; ***P < 0.001. [file 13567_2025_1586_MOESM4_ESM.tif]

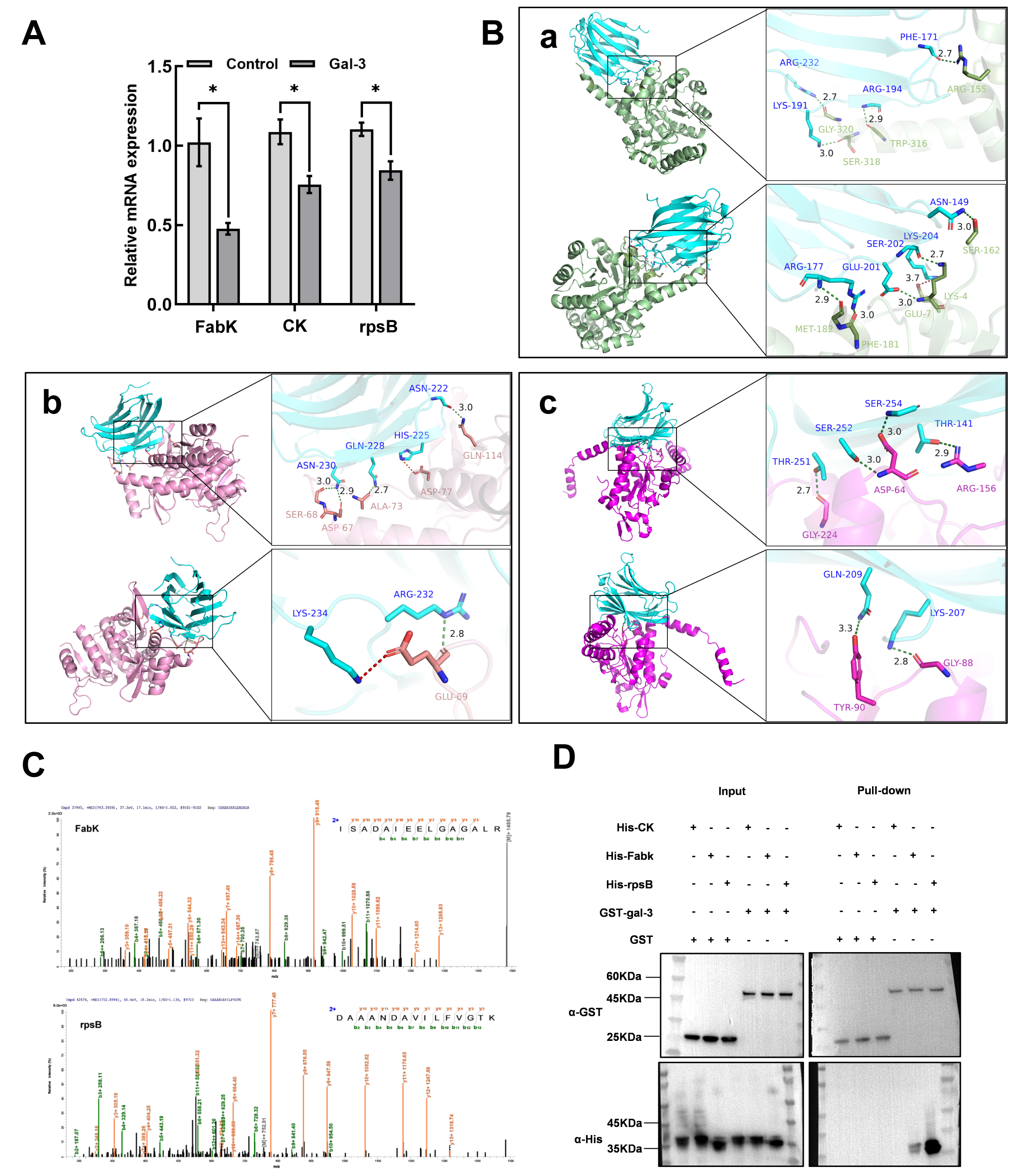

Supplement: Supplementary file 6 — Additional file 6. Galectin-3 interacts with FabK and rpsB. A RT-qPCR analysis of mRNA expression levels of FabK, CK, and rpsB in S. suis treated with galectin-3 for 2 h. B Molecular docking predictions of galectin-3 interactions with FabK (a), CK (b), and rpsB (c), respectively. C Ni-NTA magnetic beads were used to enrich streptococcal cell-associated proteins interacting with His-galectin-3, followed by identification of FabK and rpsB through nano-LC-ESI-MS/MS analysis. The secondary mass spectrum of FabK and rpsB confirms their specific binding to His-galectin-3. D GST pull-down assays were performed using GST-galectin-3. His-CK, His-FabK, and His-rpsB were incubated with GST or GST-galectin-3 immobilized on glutathione-sepharose beads. Bound proteins were analyzed by SDS-PAGE and detected by western blotting. [file 13567_2025_1586_MOESM6_ESM.tif]

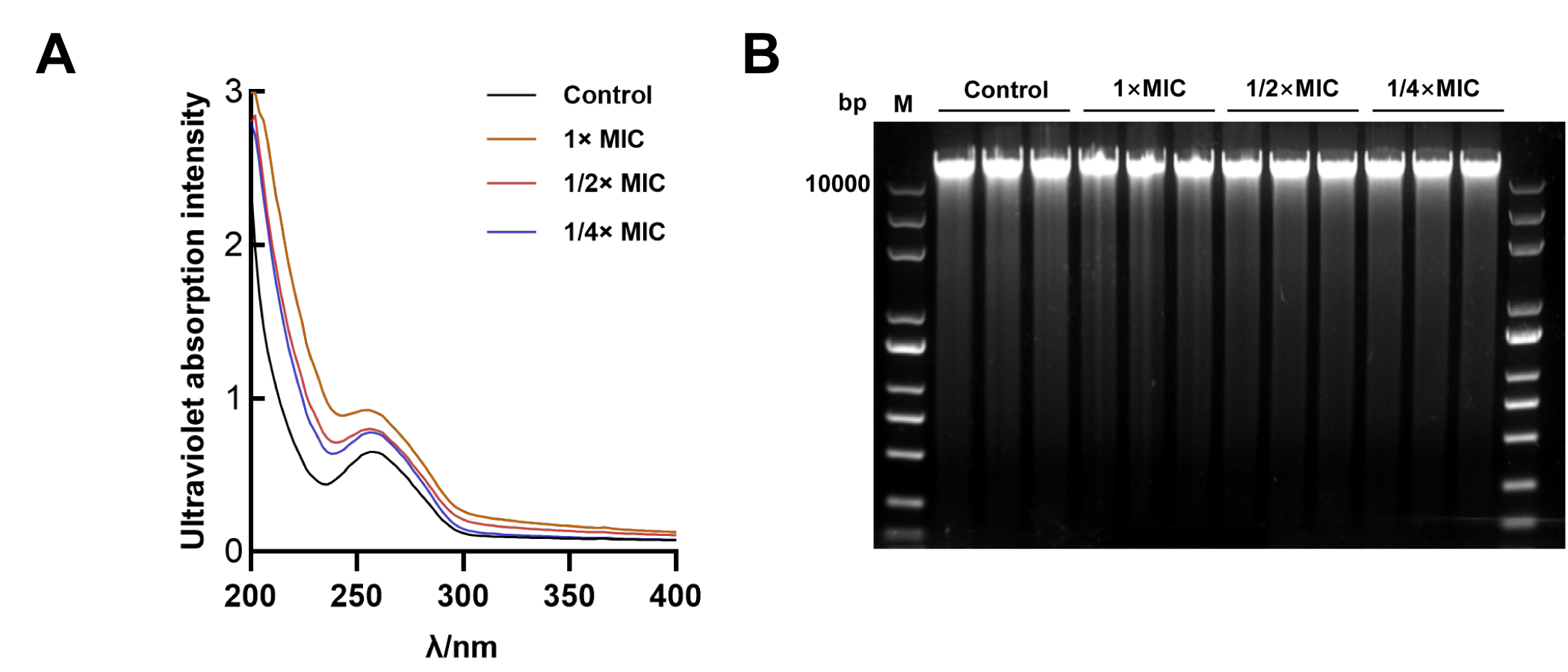

Supplement: Supplementary file 7 — Additional file 7. Galectin-3 does not interact with bacterial genomic DNA. A UV spectrum analysis of galectin-3 co-incubated with S. suis genomic DNA at different concentrations for 1 h, measured within the 200-400 nm wavelength range. B Agarose gel electrophoresis (0.7%) showing the effect of galectin-3 at different concentrations on the migration of S. suis genomic DNA after 1 h of co-incubation. [file 13567_2025_1586_MOESM7_ESM.tif]

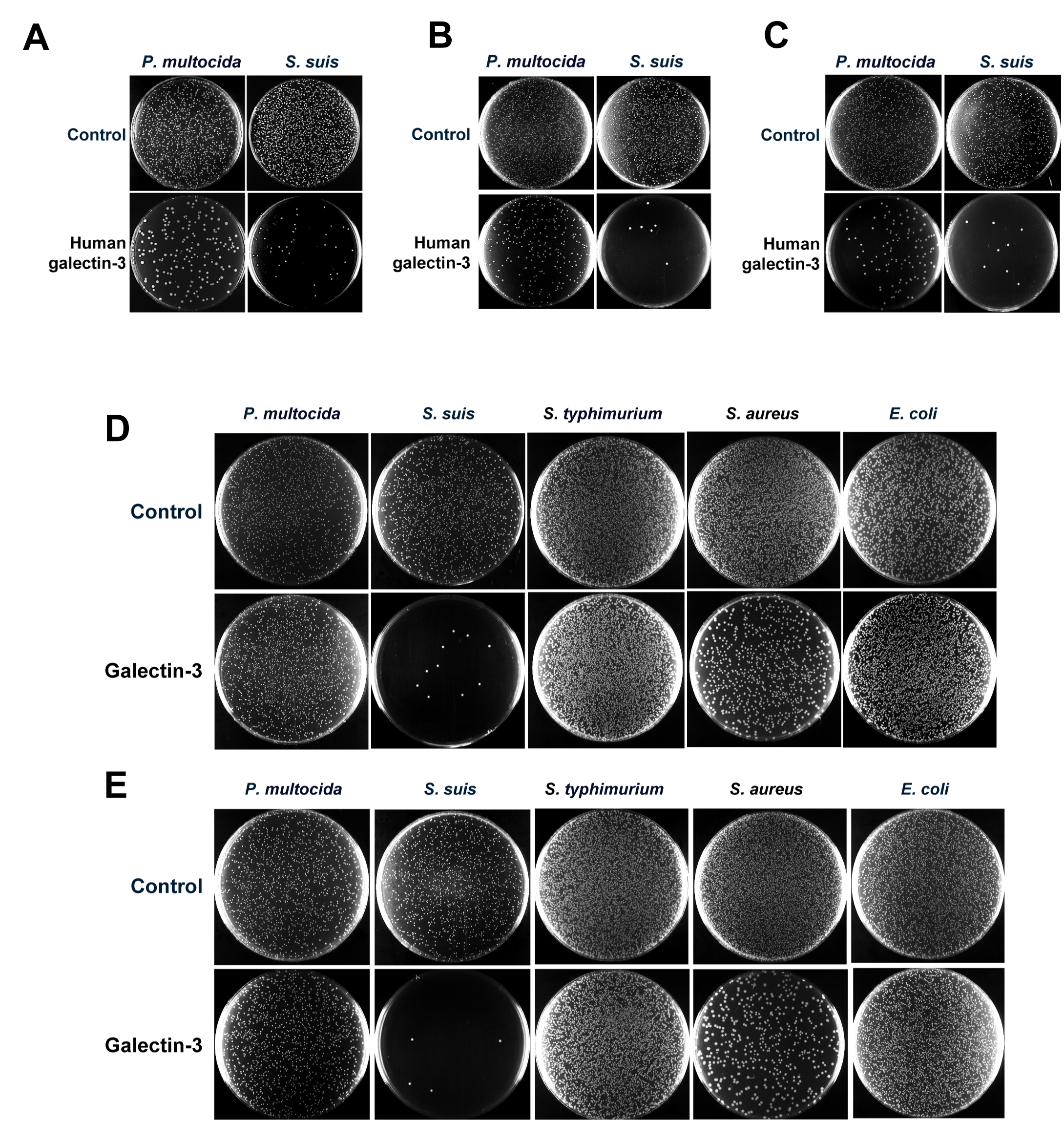

Supplement: Supplementary file 8 — Additional file 8. Replicates of bacterial colony assays. A-C Representative images of bacterial colonies on agar plates from three different experiments of Figure 2B. D-E Two additional independent experiments of bacterial colonies on agar plates from three different experiments of Figure 3A. [file 13567_2025_1586_MOESM8_ESM.tif]

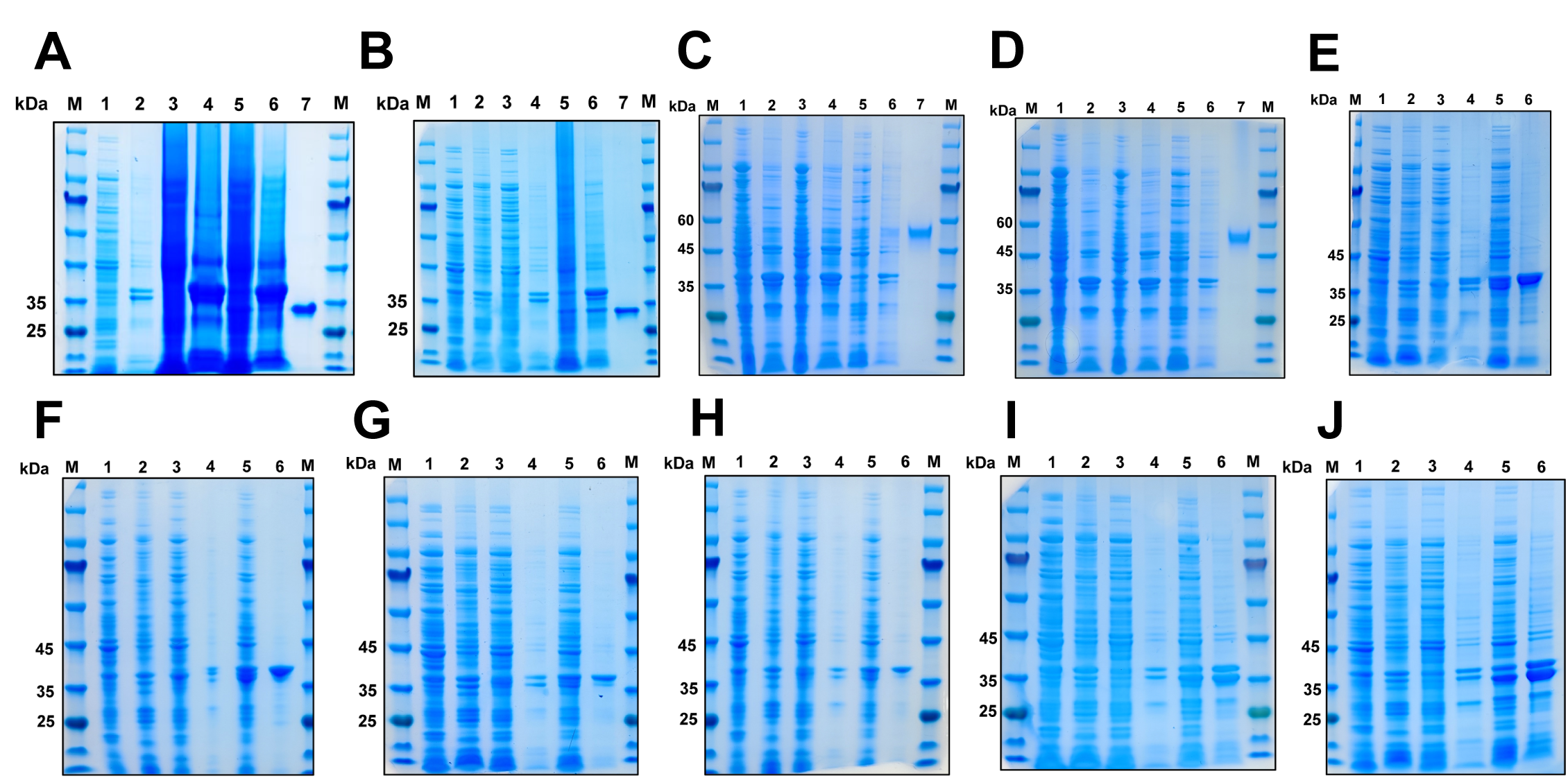

Supplement: Supplementary file 9 — Additional file 9. SDS-PAGE of protein expression replicates. The SDS-PAGE results from the two additional independent experiments of protein expression for His-galectin-3 (A and B), GST-galectin-3 (C and D), His-CK (E and F), His-FabK (G and H), and His-rpsB (I and J), respectively. [file 13567_2025_1586_MOESM9_ESM.tif]

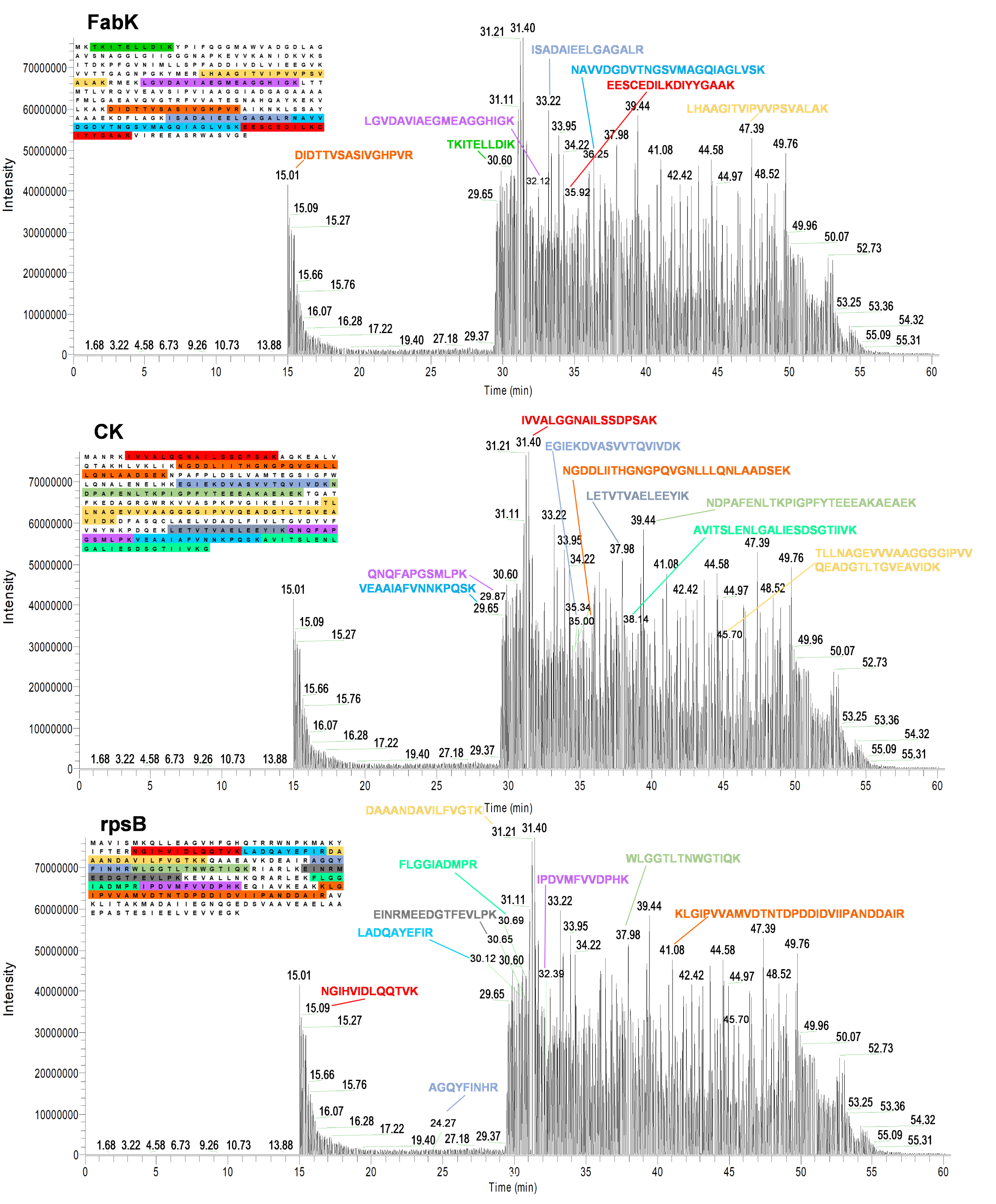

Supplement: Supplementary file 10 — Additional file 10. The total ion chromatogram (TIC) profile of tryptic digestion peptides. The TIC shows the mass spectrometry peak profile of the sample following tryptic digestion, with annotated peaks corresponding to the identified peptides in Figure 7C. [file 13567_2025_1586_MOESM10_ESM.tif]

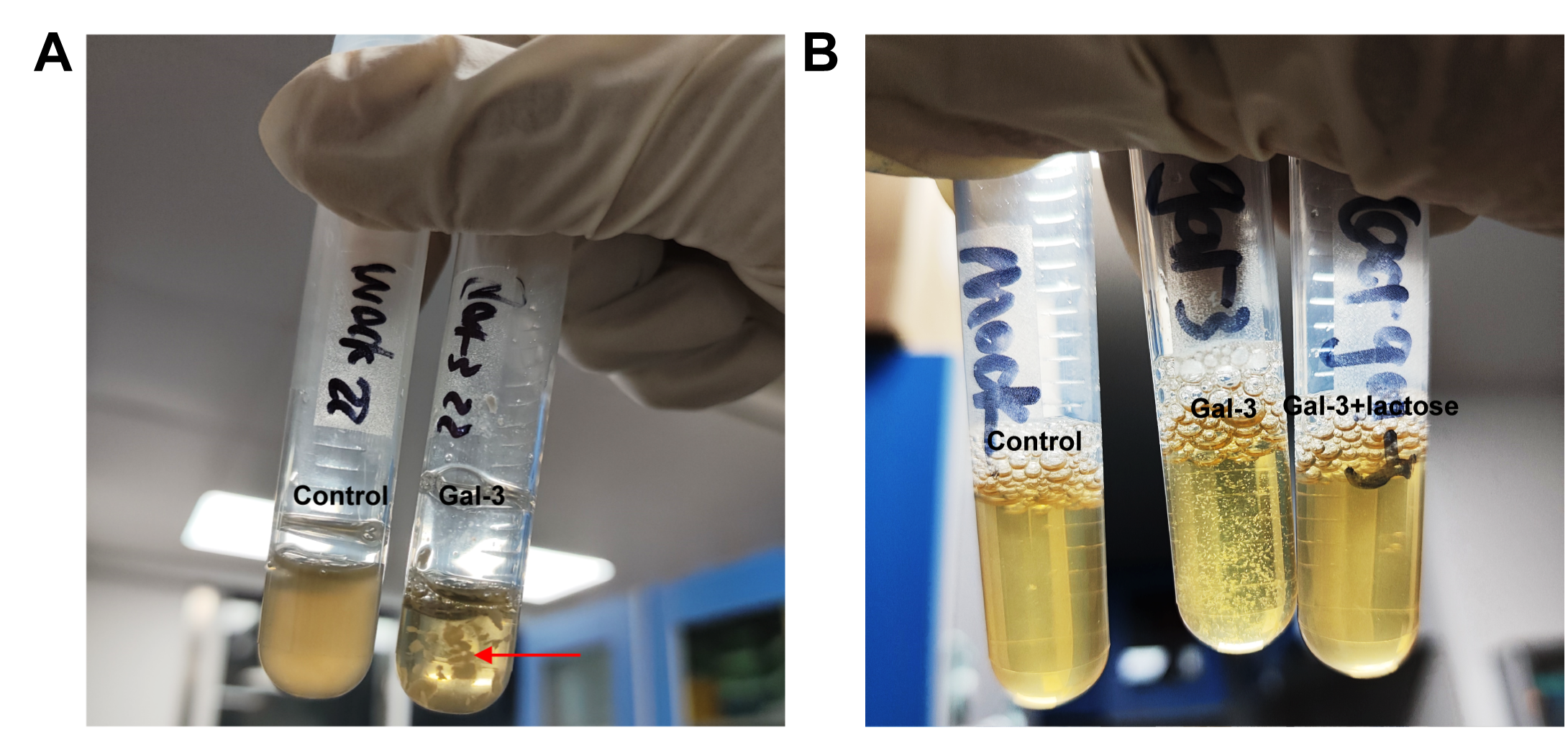

Supplement: Supplementary file 11 — Additional file 11. Visual evidence of bacterial aggregation. A Images showing bacterial aggregation and sedimentation in test tubes following galectin-3 treatment. B Images demonstrating that lactose addition inhibited galectin-3-induced aggregation and sedimentation of S. suis. [file 13567_2025_1586_MOESM11_ESM.tif]
