# Supplementary material for: Galectin-3: a novel antimicrobial host factor identified in goat nasal mucus
Source: Vet Res. 2025 Jul 21;56:153. doi: 10.1186/s13567-025-01586-w (PMC12281822; doi:10.1186/s13567-025-01586-w)
Supplement: Supplementary file 5 — Additional file 5. Summary of proteins identified after galectin-3 treatment of S. suis. [file 13567_2025_1586_MOESM5_ESM.docx]

**Additional file 5. Summary of proteins identified after galectin-3 treatment of *S. suis***.

| Protein Mass | No. of Peptide | Sequence Header | Accession | Relative Abundance | Probability | Score | SI | AA No | SIn*1E6 | No. of Unique Peptide |
| --- | --- | --- | --- | --- | --- | --- | --- | --- | --- | --- |
| 33047.39 | 50 | >tr\|A0A0H3N1F3\|A0A0H3N1F3_STRS4 Enoyl-ACP reductase OS=Streptococcus suis (strain BM407) OX=568814 GN=fabK PE=4 SV=1 | A0A0H3N1F3 | 13.0% | 99.0% | 719 | 3806695 | 321 | 425.2763657 | 8 |
| 33478.56 | 30 | >tr\|A0A075SIV4\|A0A075SIV4_STRSU Carbamate kinase OS=Streptococcus suis 6407 OX=1214179 GN=ID09_06895 PE=3 SV=1 | [A0A075SIV4](http://www.uniprot.org/uniprot/A0A075SIV4) | 12.2% | 99.0% | 458 | 3496406 | 315 | 398.0517171 | 14 |
| 28590.02 | 25 | >sp\|A4W453\|RS2_STRS2 Small ribosomal subunit protein uS2 OS=Streptococcus suis (strain 98HAH33) OX=391296 GN=rpsB PE=3 SV=2 | [A4W453](http://www.uniprot.org/uniprot/A4W453) | 14.8% | 99.0% | 378 | 3469153 | 258 | 482.2052636 | 12 |
| 36912.16 | 15 | >tr\|A0A0K2E684\|A0A0K2E684_STRSU Ribonucleoside-diphosphate reductase subunit beta OS=Streptococcus suis OX=1307 GN=nrdB PE=3 SV=1 | [A0A0K2E684](http://www.uniprot.org/uniprot/A0A0K2E684) | 4.8% | 99.0% | 235 | 1400986 | 319 | 157.4966465 | 11 |
| 33268.91 | 14 | >tr\|G7SEJ9\|G7SEJ9_STRSU Probable manganese-dependent inorganic pyrophosphatase OS=Streptococcus suis D12 OX=1004952 GN=ppaC PE=3 SV=1 | [G7SEJ9](http://www.uniprot.org/uniprot/G7SEJ9) | 6.7% | 99.0% | 199 | 1886455 | 311 | 217.5275455 | 7 |
| 35447.67 | 13 | >tr\|A0A0H3MX19\|A0A0H3MX19_STRS4 Pyruvate dehydrogenase E1 component subunit beta OS=Streptococcus suis (strain BM407) OX=568814 GN=pdhB PE=4 SV=1 | [A0A0H3MX19](http://www.uniprot.org/uniprot/A0A0H3MX19) | 4.3% | 99.0% | 193 | 1301242 | 331 | 140.9802503 | 8 |
| 34798.88 | 13 | >tr\|A0A075SJI7\|A0A075SJI7_STRSU HPr kinase/phosphorylase OS=Streptococcus suis 6407 OX=1214179 GN=hprK PE=3 SV=1 | [A0A075SJI7](http://www.uniprot.org/uniprot/A0A075SJI7) | 3.7% | 99.0% | 208 | 1038330 | 310 | 120.1162892 | 9 |
| 30857.12 | 12 | >tr\|A0A0K2E893\|A0A0K2E893_STRSU Amino acid ABC transporter substrate-binding protein OS=Streptococcus suis OX=1307 GN=yxeM PE=4 SV=1 | [A0A0K2E893](http://www.uniprot.org/uniprot/A0A0K2E893) | 4.8% | 99.0% | 206 | 1241468 | 283 | 157.3175929 | 7 |
| 44019.41 | 10 | >tr\|A0A075SFE8\|A0A075SFE8_STRSU Elongation factor Tu OS=Streptococcus suis 6407 OX=1214179 GN=tuf PE=3 SV=1 | [A0A075SFE8](http://www.uniprot.org/uniprot/A0A075SFE8) | 2.3% | 99.0% | 165 | 846089 | 398 | 76.23618854 | 7 |
| 34960.35 | 8 | >tr\|A0A075SLB5\|A0A075SLB5_STRSU Glycine--tRNA ligase alpha subunit OS=Streptococcus suis 6407 OX=1214179 GN=glyQ PE=3 SV=1 | [A0A075SLB5](http://www.uniprot.org/uniprot/A0A075SLB5) | 1.8% | 99.0% | 130 | 491702 | 305 | 57.8136427 | 6 |
